# Switching between Hydrogenation and Olefin Transposition Catalysis via Silencing NH Cooperativity in Mn(I) Pincer Complexes

**DOI:** 10.1021/acscatal.2c02963

**Published:** 2022-08-19

**Authors:** Wenjun Yang, Ivan Yu. Chernyshov, Manuela Weber, Evgeny A. Pidko, Georgy A. Filonenko

**Affiliations:** † Inorganic Systems Engineering Group, Department of Chemical Engineering, Faculty of Applied Sciences, Delft University of Technology, Van der Maasweg 9, 2629 HZ Delft, The Netherlands; ‡ TheoMAT Group, ChemBio Cluster, ITMO University, Lomonosova 9, St. Petersburg 191002, Russia; § Institute of Chemistry and Biochemistry, Freie Universität Berlin, Fabeckstraße 34/36, D-14195 Berlin, Germany

**Keywords:** olefin transposition, manganese complex, metal−ligand cooperation, metal hydrides, ligand dynamics, N−H functionality

## Abstract

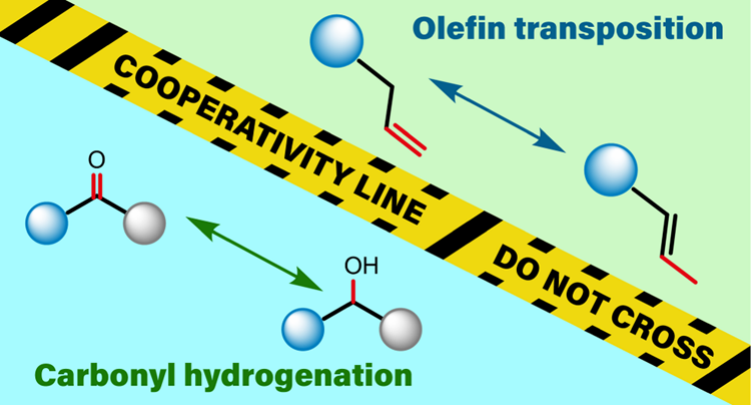

While Mn-catalyzed (de)hydrogenation of carbonyl derivatives has been well established, the reactivity of Mn hydrides with olefins remains very rare. Herein, we report a Mn(I) pincer complex that effectively promotes site-controlled transposition of olefins. This reactivity is shown to emerge once the N–H functionality within the Mn/NH bifunctional complex is suppressed by alkylation. While detrimental for carbonyl (de)hydrogenation, such masking of the cooperative N–H functionality allows for the highly efficient conversion of a wide range of allylarenes to higher-value 1-propenybenzenes in near-quantitative yield with excellent stereoselectivities. The reactivity toward a single positional isomerization was also retained for long-chain alkenes, resulting in the highly regioselective formation of 2-alkenes, which are less thermodynamically stable compared to other possible isomerization products. The detailed mechanistic analysis of the reaction between the activated Mn catalyst and olefins points to catalysis operating via a metal–alkyl mechanism—one of the three conventional transposition mechanisms previously unknown in Mn complexes.

Carbon–carbon double bonds are key skeletal units in a plethora of natural and industrial chemicals^1^ as well as versatile precursors for many synthetic transformations.^2^ Despite the availability of numerous protocols for installing olefin functional groups (e.g., olefination, elimination, condensation, dehydrogenation), such transformations are frequently disadvantaged by low stereoselectivities or restrictions on functional groups.^3^ Alternatively, transposition of pre-existing olefins offers a powerful and atom-economical route to incorporate and manipulate C=C bonds with far-reaching applications in industrial production, e.g., pharmaceuticals, cosmetics, fragrances, polymers, and fuels.^1b,4^ Various processes were developed with efficient catalysts based on transition metals: Ir,^5^ Rh,^6^ Pd,^7^ Ru,^8^ Cr,^9^ Co,^10^ Ni,^11^ Fe,^12^ and W,^13^ among others (Figure 1a). Missing in this set of examples is the highly abundant and biocompatible manganese metal that remains unknown in olefin transposition so far.

**Figure 1:**
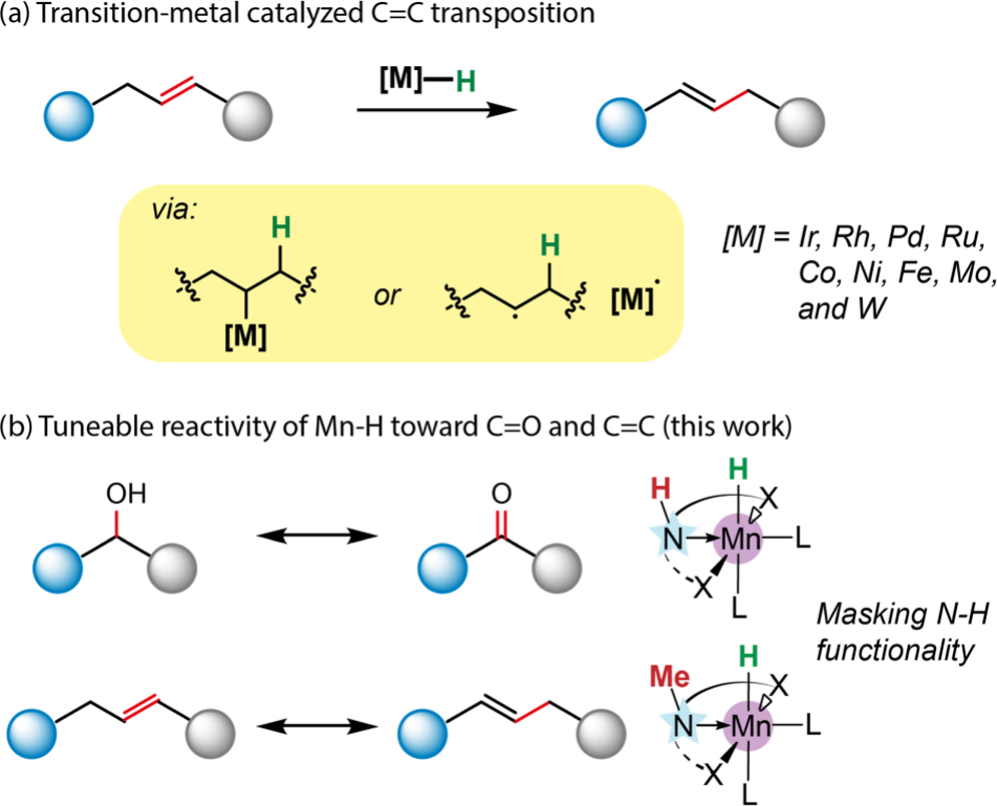
Metal complex-mediated olefin transpositions (a) and the reactivity discussed in this work (b).

Alkene transposition catalysis is mechanistically diverse and generally proceeds via either of the three alternative paths, namely, allyl, alkyl, or radical mechanisms with the latter two governing the activity of the vast majority of catalyst systems.^4c,10i,14^ In both mechanisms, metal hydrides are the active species, which promote the transposition reaction via H^–^/H^•^ addition to the alkene, followed by β-H elimination/H^•^ abstraction to furnish the isomerization product.^4c^

The representative examples operating via the alkyl mechanism such as Pd(dba)_2_ (Skrydstrup),^7a^ Co–NNP complexes (Liu),^10f^ and Fe(OAc)_2_ (Koh)^12b^ typically require in situ activation to form catalytic metal hydride species by the reaction with reagents such as acyl chloride, ammonia borane, and boryl reagent combined with a base. Activation-free olefin transposition catalysis was also demonstrated with isolated metal hydride or metal–alkyl complexes.^10b,12c^ With respect to radical-type processes, the latest advances were disclosed by Shenvi and Palmer groups employing cobalt salen^10c^ and cobaloxime complexes,^10e^ respectively. Upon the reductive treatment, these complexes form Co hydrides that can act as H^•^-donors. The central role of metal hydrides for the catalytic C=C bond suggests a potentially broader scope of catalyst systems for this chemistry.

Manganese complexes emerged as potent carbonyl (de)hydrogenation catalysts in the last decade together with other two base metals, Fe and Co.^15^ The generation of Mn hydride species has been widely accepted as a prerequisite for the (de)hydrogenation cycle.^16^ However, the hydride transfer to nonpolar olefins remains uncommon for Mn homogeneous catalysis. Recently, a few cases of alkene hydrogenations have been reported with Mn non-pincer complexes.^17^ In particular, the alkyl bisphosphine Mn(I) catalyst reported by the Kirchner group forms active 16e Mn hydride under a H_2_ atmosphere that can reduce a range of mono- and disubstituted alkenes to alkanes.^17a^

Given these results, we envisioned that the olefin transposition reactivity could be accessible by Mn-based systems. Herein, we disclose that by masking the metal/NH cooperativity, one can tune the reactivity of Mn hydrides from polar C=X (X = O, N) substrates to C=C bonds. With this strategy, we develop the first highly selective olefin transposition reactions catalyzed by Mn(I), specifically an N-methylated Mn–CNP complex (Figure 1b).

At the onset of the investigation, we screened the activity of several well-defined Mn(I) complexes reported by our group and others (Figure 2) toward the transposition of the model substrate 4-allylanisole (**1a**). Precatalysts **Mn-3–5**,^16j,18^ which were reported to be efficient for carbonyl hydrogenation, surprisingly displayed no reactivity in transposition with the exception of **Mn-3**, which gave 18% yield of isomerized product **2a** (Table 1, entries 1–3).

**Figure 2:**
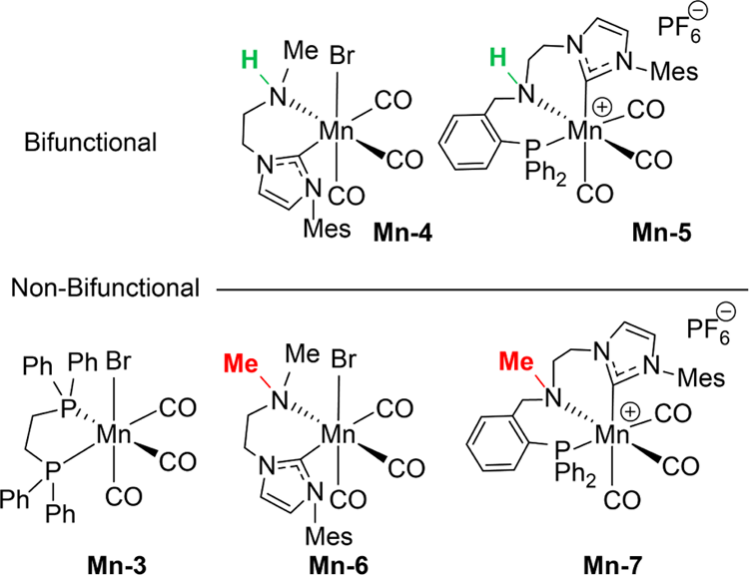
Mn catalysts used in this study.

**Table 1:**
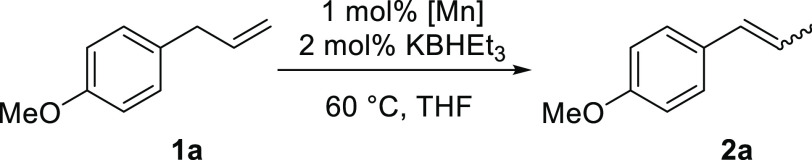
Manganese-Catalyzed Transposition of the Model Compound 4-Allylanisole^a^

entry	[Mn]	time (h)	yield (%)	*E*:*Z*
1	Mn-3	12	18	88:12
2	Mn-4	12	trace	
3	Mn-5	12	trace	
4	Mn-6	12	27	86:14
5	Mn-7	12	61	85:15
6	Mn-7	24	89 (89)^b^	91:9
7		24	trace	
8^c^	Mn-7	24	trace	

a Reaction conditions: **1a** (0.25 mmol), Mn catalyst (1 mol %), and 2 mol % KBHEt_3_ in 0.5 mL of THF at 60 °C.

b Conversion is given in parenthesis.

c KBHEt_3_ is not used for the activation of the Mn catalyst.

We assumed the N–H functionality might be detrimental to C=C transposition and synthesized N–H methylated complexes **Mn-6** and **Mn-7** based on **Mn-4** and **Mn-5** (see the Supporting Information for synthetic details). Interestingly, once N–H functionality is blocked, Mn complexes start exhibiting the transposition activity (entries 4 and 5), with **Mn-7** giving the highest yield in the model reaction (61%). The yield and *E*-selectivity in product **2a** could be increased to 89% and 91:9 (*E*/*Z*) in a prolonged run (entry 6). Further screening of solvents and reaction temperatures confirmed the THF solvent and 60 °C temperature to be optimal for the catalytic performance of **Mn-7** (see Table S1). Control experiments indicated the necessity of the catalyst activation with KBHEt_3_, which typically allows for a more selective generation of Mn hydrides (entries 7 and 8).

With the transposition reactivity established, we sought to examine the generality of this process. A broad scope of substrates can be converted with good selectivities with complex **Mn-7** (Scheme 1). Industrially relevant anethole, isoeugenol, isosafrole, and isoelemicin (**2a–2d**) were successfully generated via the transposition reaction in excellent yields (72–99%) and *E*/*Z* ratios (>90:10). Our protocol is also efficient toward allylbenzene (**1e**) and its substituted derivatives with electron-withdrawing groups (**1f**–**1h**), electron-donating groups (**1i**–**1m**), and sterically hindered naphthyl (**1n**), furnishing the desired styrenyl products in ≥91% yields and ≥92% *E* selectivities. Substrates containing heterocycles (**1o** and **1p**) and carbonyl groups (**1q**) were tolerated but converted in lower yields and stereoselectivities.

**Scheme 1:**
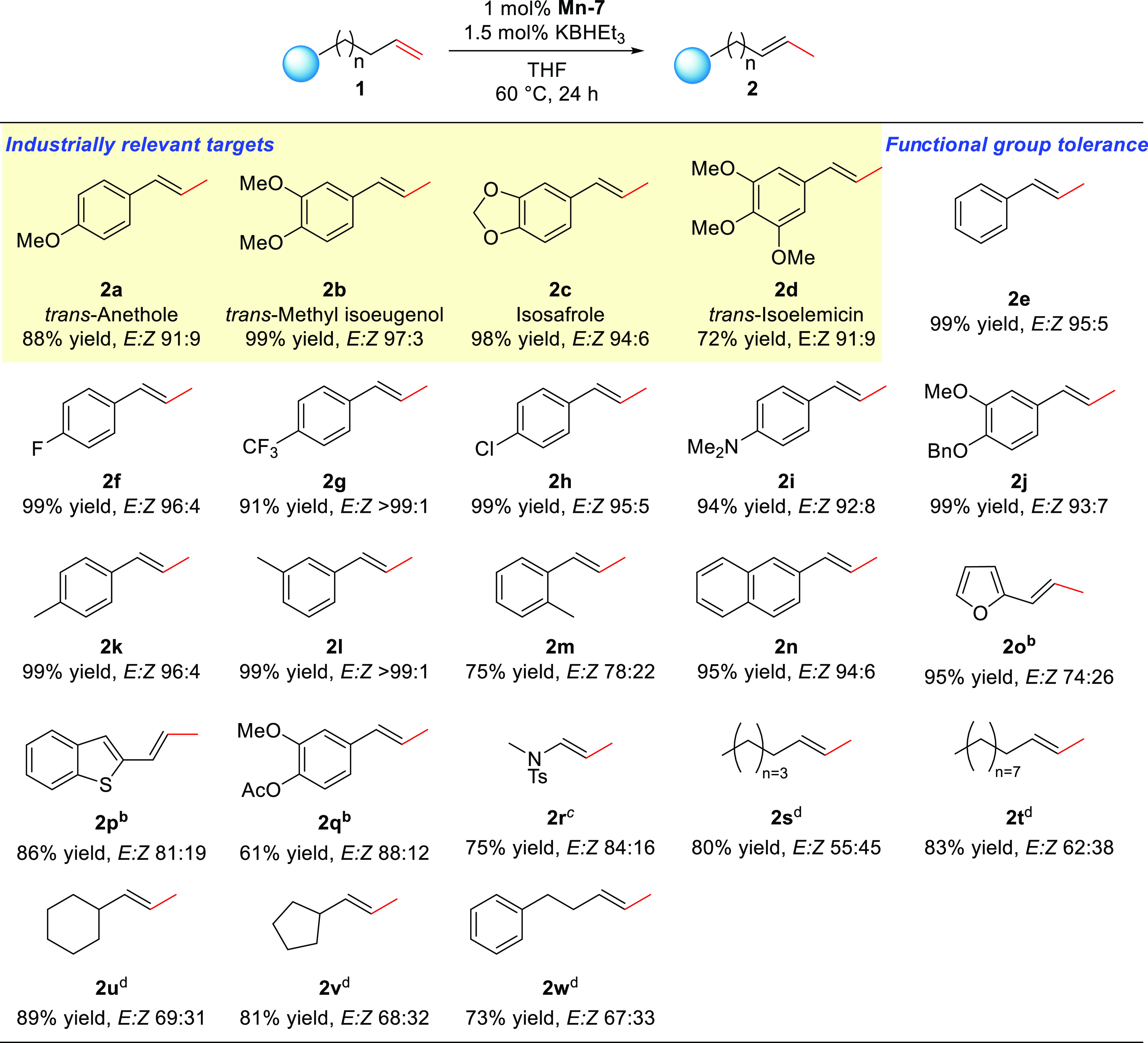
Catalytic Double-Bond Transposition with **Mn-7** Reaction conditions: substrate **1** (0.25 mmol), **Mn-7** (1 mol %), and 2 mol % KBHEt_3_ in 0.5 mL of THF at 60 °C for 24 h. Reaction was performed with 4 mol % **Mn-7** at 70 °C. 5 mol % **Mn-7** was used instead. Reaction was performed with 4 mol % **Mn-7** at 70 °C in toluene instead.

Controlling the site selectivity is a recognized challenge for migrating the C=C bonds over extended carbon skeletons, due to the thermodynamic similarities of positionally isomerized products. **Mn-7** allows for the highly regioselective monoisomerization of long-chain alkenes, even though further migration could be thermodynamically more favorable. Both 1-octene (**1s**) and 1-dodecene (**1t**) were isomerized to corresponding 2-alkenes in excellent yield, albeit with moderate *E*/*Z* ratios. The monoisomerization process is also compatible with functionalities (**1u–1w**), including cycloalkyl and phenyl.

The N–H functionality has been broadly reported as the key structural parameter that enables the (de)hydrogenation of polar moieties and Mn/NH bifunctional behavior in principle.^19^ This was typically confirmed in the studies where alkylation of the N–H functionality produced inactive (de)hydrogenation catalysts.^16h,16i,18a,20^ Our catalytic data (Table 1, entries 2–5) implies that for olefin transposition, this structure–activity relationship is inverted.

To confirm this, we compared the C=O/C=C substrate preference using cooperative and noncooperative Mn(I)–CNP counterparts: **Mn-5** and **Mn-7**, respectively. As depicted in Figure 3, the N–H methylation in Mn-CNPs completely suppressed the ketone hydrogenation but enabled the transposition of allylbenzene and even the hydrogenation of styrene. Notably, **Mn-5** with the cooperative N–H functionality was inactive for either of the C=C bond transformation paths. The displayed selectivity prompted further mechanistic analysis of **Mn-7** operation in the course of the reaction.

**Figure 3:**
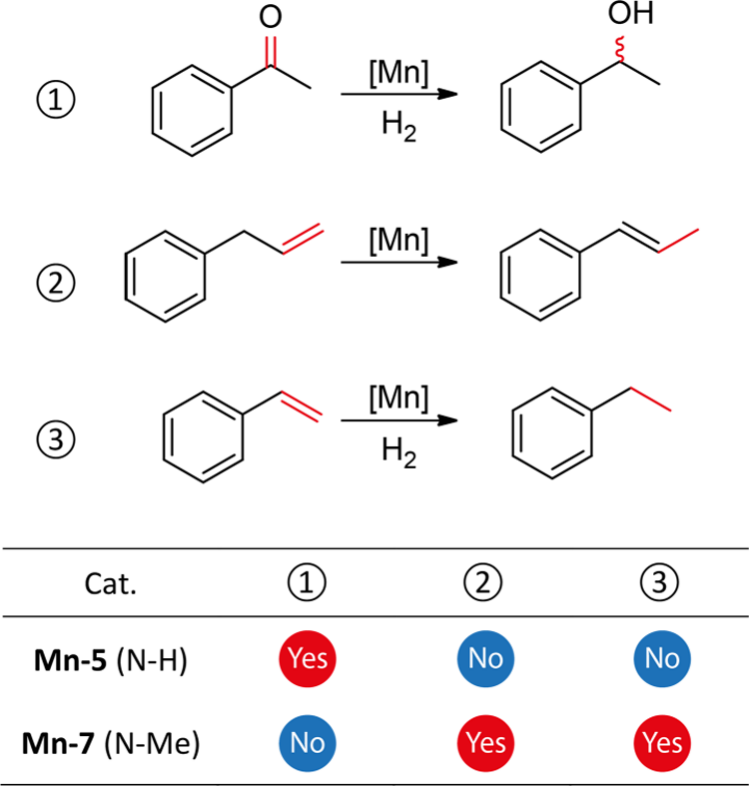
Reactivities of Mn(I)–CNP complexes toward C=O and C=C functionalities. See Section S7 for reaction details.

As a first step in the mechanistic investigation, we conducted cross-reactivity experiments with deuterium-labeled **11-d** and nondeuterated **2a** (Scheme 2). We observed both the intramolecular scrambling as well as the intermolecular crossover of the deuterium label between the olefin products. This is indicative of transposition proceeding via either alkyl or hydrogen atom transfer mechanisms because the cross-reactivity between deuterated and label-free olefins should involve a Mn–H species as a transfer medium. Together with the previous observation that KHBEt_3_ activation was necessary for the catalytic reactivity (Table 1, entry 8), labeling data implies that the formation of Mn hydride must take place in the course of the reaction. To verify this, we monitored the KBHEt_3_ activation of precatalyst **Mn-7** followed by the catalytic turnover using NMR and IR spectroscopy.

**Scheme 2:**
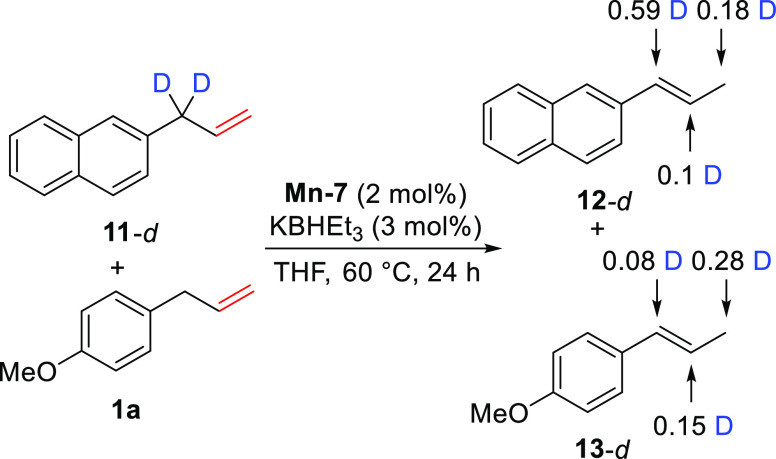
Results of Deuterium Crossover Experiments

**Mn-7** readily forms hydrides upon activation. At room temperature, the reaction of **Mn-7** with KBHEt_3_ in THF-*d*_8_ gave rise to three new doublet resonances in the ^1^H NMR spectrum at −5.01, −5.65, and −6.77 ppm with ^2^*J*_PH_ = 40.0, 48.0, and 88.0 Hz, respectively (Figure 4a,b). We attributed these peaks to the isomers of tricarbonyl Mn–H species **8**, in total accounting for 97% of the activation products. The retention of three CO ligands upon the near-quantitative transformation of **Mn-7** during the activation step is confirmed by IR spectroscopy revealing three new bands at 1981, 1896, and 1876 cm^–1^ (Figure 4c). Since **8** is a tricarbonyl monohydride complex featuring a Mn-bound phosphine donor, we conclude that activation of **Mn-7** leads to the dissociation of the central N-donor group, rendering it hemilabile (see Section S8 for the structural assignments based on the exhaustive expert-bias-free configurational DFT analysis). This contrasts the case of nonmethylated analogue **Mn-5**,^16j^ which dissociated the phosphine arm in a similar activation step.

**Figure 4:**
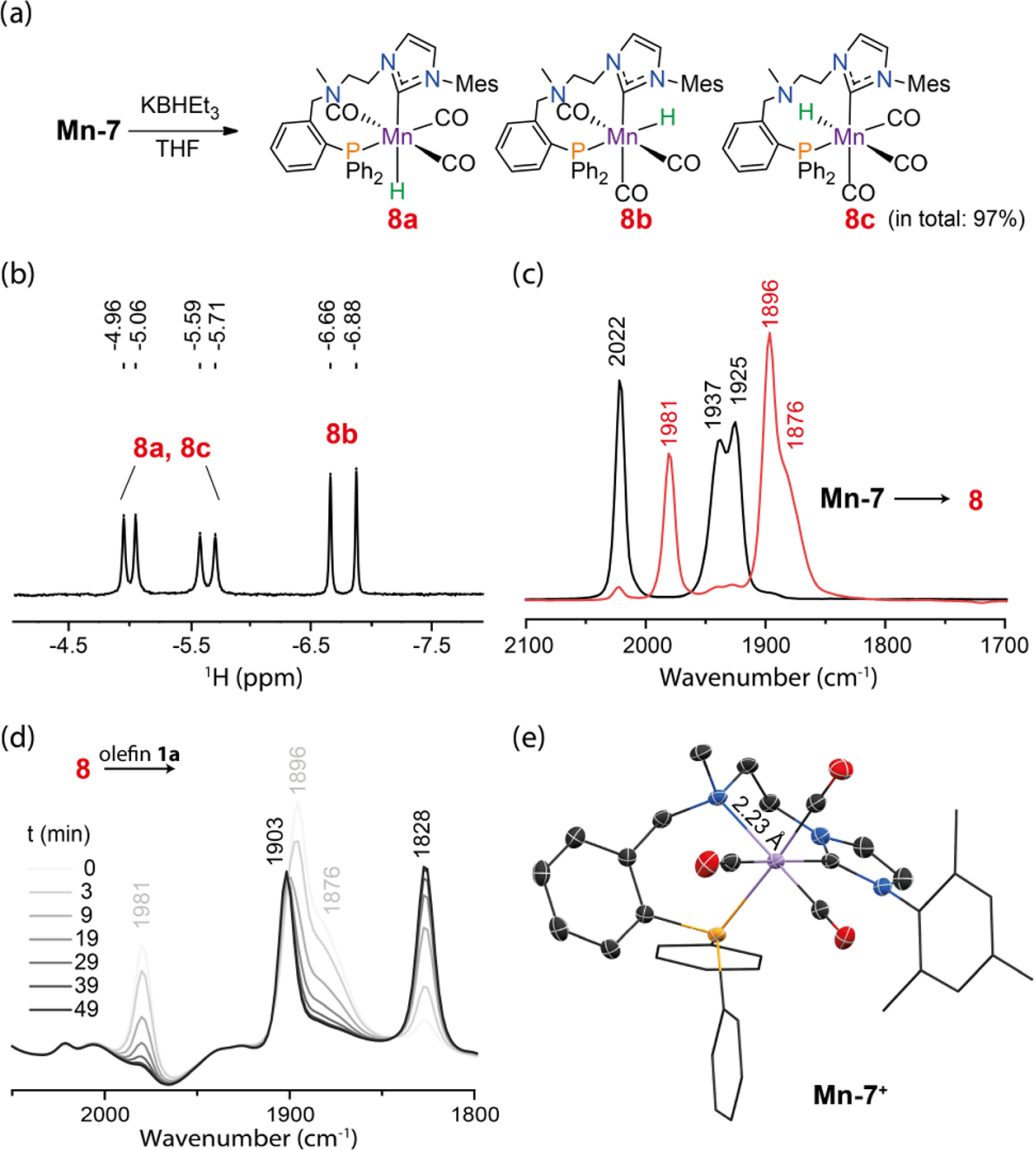
Activation of **Mn-7** upon the reaction with KBHEt_3_ (a). The hydride region of the ^1^H NMR (THF-*d*_8_) spectra (b) of in situ generated complex **8** (see Figure S11 for full spectra) and the IR spectra (c) of complex **Mn-7** (black) and in situ generated complex **8** (red) recorded in THF. Time-dependent IR spectra evolution (d) for the reaction of complex **8** with olefin (0–49 min, gray to black). Molecular structure (e) of complex **Mn-7** in the solid state with thermal ellipsoids drawn at 50% probability. Hydrogen atoms are omitted for clarity.

The dissociation of the central N-donor is the major transformation within the CN(Me)P ligand upon the activation, and the hydride complex with the dissociated P-donor was observed in minor amounts (3% NMR yield, see Figure S11). The difference between Mn hydrides with the dissociated N- or P-donor is reflected in hydride ligand shifts in ^1^H NMR (Figure S11) and is supported by DFT calculations (Table S4). Based on DFT analysis, we assume that complex **8** might exist as an octahedral complex with facially bound P- and C-donor groups with isomers distinguished by hydride ligand placement *trans* to NHC, phosphine, or carbonyl ligand (**8a**, **8b**, and **8c**, respectively, Figure 4); all three featuring the dissociated central N-donor group.

Hydride complexes **8** readily react with olefins. A clean consumption of hydrides in **8** was observed within minutes upon the addition of 4-allylanisole (**1a**). The analysis of this reaction with IR and NMR spectroscopy indicates the formation of the dicarbonyl Mn–alkyl species. Namely, the IR spectrum (Figure 4d) indicates the consumption of **8** and the formation of two new bands at 1903 and 1828 cm^–1^ typical of dicabonyl complexes. In the absence of the hydride resonance in the NMR spectrum, this suggests that the reaction of **8** with olefin leads to the formation of metal–alkyl complexes with the N-donor group reattaching to the Mn center. As expected, the gradual production of **2a** was then detected in ^1^H NMR upon heating of the reaction mixture to 60 °C (see Figure S16). This observation of hydride transfer suggests that **Mn-7** isomerizes olefins via the alkyl mechanism. Since we observe no further change in the NMR spectrum, we assume that the metal–alkyl complexes likely represent the resting states in this transformation as was earlier proposed for the high-spin cobalt(II) system by Wiex, Holland, and co-workers.^10b^ The transformation that follows the resting state formation in catalysis is typically the rate-determining step. The rate of the olefin transposition reaction should then be determined by the rate of β-hydride elimination (Figure 5, intermediates **II** and **III**), suggesting that the catalysis rate should be independent of substrate concentration. Indeed our kinetic studies revealed an order of 0.14 with respect to the alkene substrate, confirming the hypothesis above (see Figure S17).

**Figure 5:**
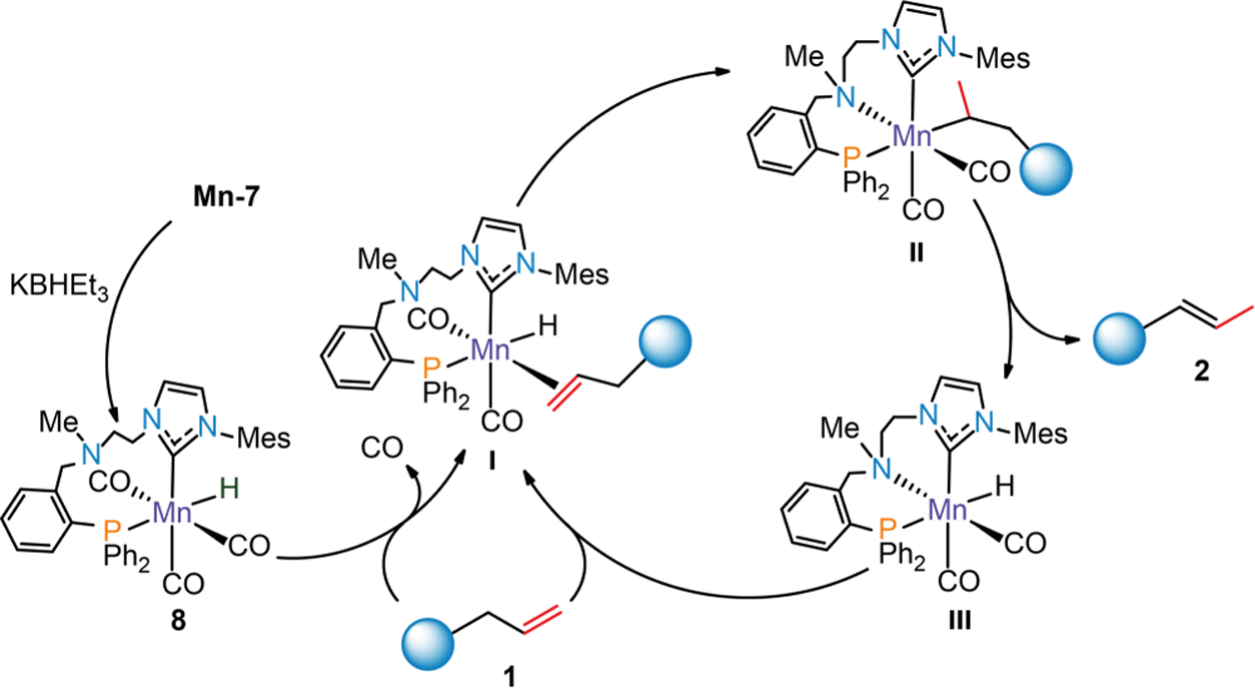
Mechanistic proposal for Mn-catalyzed olefin transpositions.

Based on the results above, we conclude that the metal–alkyl mechanism is likely operating in the present catalytic system (Figure 5). Activated Mn(I) hydride precatalyst **8** enters the cycle via the reaction with alkene via intermediate **I**. This step requires the dissociation of a CO ligand detected experimentally. Further transformation of **I** involves the hydride transfer to form Mn–alkyl species **II** aided by the reattachment of the N-donor ligand. Subsequent β-hydride elimination furnishes isomerized olefin product **2** and dicarbonyl Mn hydride **III**. The final coordination of another alkene substrate can be kinetically unfavorable due to the saturation of the Mn center with strong field ligands. However, we speculate that this step can be facilitated by the dissociation of the labile N-donor within the Mn–CN(Me)P complex that would liberate the vacant site for olefin coordination, regenerating species **I**. In support of this suggestion, **Mn-7** shows significantly inhibited activity in the presence of competing ligands, e.g., CO and PPh_3_, that might compete for vacant sites or inhibit the first CO dissociation step (Table 2).

Similar to the case of NH-cooperative Mn(I) catalyst **Mn-5**, our results reveal the high extent of tridentate ligand dynamics throughout the catalyst activation. The suggested involvement of N-donor dissociation in the catalytic cycle^21^ would rationalize the selectivity flip toward C=C for Mn complexes obtained by blocking the N–H functionality (Table 1 and Figure 3). To investigate the occurrence of ligand dissociation, control experiments of transposition catalysis were performed in the presence of a series of donor additives that can compete with olefin substrates for the open sites of Mn catalysts (Table 2). The additions of strong field ligands, PPh_3_ and CO, both rendered **Mn-7** nearly inactive (entries 2 and 3), while the reactions with weaker field ligands, pyridine and acetonitrile, gave much lower yields of **2a** (entries 4 and 5) compared to the additive-free experiment (entry 1). The inhibitory effects of donor additives validate the importance of generating coordination space for catalysis. Analyzing the crystal structures (Figure 4e and Table 3) of methylated **Mn-6** and **7**, we find that Mn–N bond lengths are significantly longer in these complexes compared to their NH counterparts **Mn-4** and **-5**.^16j,18a^ This trend further suggests that conventional pincer and tridentate ligands in Mn(I) complexes might exhibit dynamics and donor ligand lability that are not characteristic for their noble metal-based counterparts. While the catalytic functionality of this behavior is open to debate, it clearly invites further research into ligand dissociation dynamics of catalytically relevant Mn(I) complexes.

**Table 2:** Manganese-Catalyzed Transposition of 4-Allylanisole **1a** in the Presence of Donor Additives^a^

entries	donors	yield (%)	*E*:*Z*
1	none	89	91:9
2	PPh_3_	7	
3	CO (3 bar)	4	
4	pyridine	68	85:15
5	acetonitrile	24	83:17

a Reaction conditions: **1a** (0.25 mmol), donor additive (0.125 mmol), **Mn-7** (1 mol %), and 2 mol % KBHEt_3_ in 0.5 mL of THF at 60 °C for 24 h.

**Table 3:** Bond Length of Mn–N for Selected Mn Complexes in the Solid State Calculated from X-ray Data

	N–H		N–Me	
complexes	Mn-4	Mn-5	Mn-6	Mn-7
Mn–N (Å)	2.14	2.14^a^	2.24	2.23

a The bond length for **Mn-5** was determined by DFT analysis previously.^16j^

In summary, this work describes the first precedent of olefin transposition catalyzed by complexes based on abundant and biocompatible Mn metal. This reactivity furnishes an array of 2-alkenes in good selectivities and yields. Importantly, this activity is obtained upon disabling the cooperative function in related Mn catalysts that show activity in carbonyl hydrogenation while being virtually inactive toward olefin conversion. We envision that such manipulation on metal–ligand cooperation modes may present a new design direction for controlling early transition metal catalysts and enabling multiple reactivity trains with minimal ligand modification.

## Notes

Dataset for this publication is available from 4TU. Research data under DOI: 10.4121/19704391.

## Supporting Information Available

Full synthetic procedure descriptions; characterization data; methodology for catalytic test and calculations (PDF)
Crystallographic data for complex **Mn-7** (CIF)
xyz coordinates of computed structures (ZIP)

## Supplementary Material

cs2c02963_si_001.pdf

cs2c02963_si_002.cif
