# Supplementary material for: Switching between Hydrogenation and Olefin Transposition Catalysis via Silencing NH Cooperativity in Mn(I) Pincer Complexes
Source: ACS Catal. 2022 Aug 19;12(17):10818–25. doi: 10.1021/acscatal.2c02963 (PMC9442580; doi:10.1021/acscatal.2c02963)
Supplement: Supplementary file 1 — cs2c02963_si_001.pdf [file cs2c02963_si_001.pdf]

SUPPORTING INFORMATION

Switching Between Hydrogenation and Olefin Transposition Catalysis via Silencing NH Cooperativity in Mn(I) pincer complexes

Wenjun Yang,[a] Ivan Yu. Chernyshov,[b] Manuela Weber,[c] Evgeny A. Pidko,*,[a] Georgy A. Filonenko*,[a]

[a] Inorganic Systems Engineering group, Department of Chemical Engineering, Faculty of Applied Sciences, Delft University of Technology, Van der Maasweg 9, 2629 HZ, Delft, The Netherlands

[b] TheoMAT Group, ChemBio cluster, ITMO University, Lomonosova 9, St. Petersburg, 191002, Russia

[c] Institute of Chemistry and Biochemistry, Freie Universität Berlin, Fabeckstraße 34/36, Berlin, D-14195, Germany

Corresponding authors: Georgy A. Filonenko (G.A.Filonenko@tudelft.nl)

Evgeny A. Pidko (E.A.Pidko@tudelft.nl)

Dataset for this publication is available from 4TU.Research data under DOI: 10.4121/19704391

Contents

S1 – General Considerations 3
S2 – Synthetic Procedures and Characterization Data. 4
S3 –Reactivity Study. 12
S4-Deuterium labelling experiments... 19
S5 – Catalysis Details. 22
S6 – Optimization of Reaction Conditions. 24
S7 – Reactivities of Mn complexes toward C=O and C=C.... 25
S8 – Computational Studies... 26
S9 – Crystal Structure Analysis Details... 30
S10 – References... 31

S1 – General Considerations

All manipulations were, unless stated otherwise, performed under inert atmosphere in an argon filled glovebox (INERT) or using standard Schlenk techniques. Anhydrous solvents (THF, DCM, toluene) were either dispensed from an Inert PureSolv solvent purification system or dried using 3/4 Å molecular sieves and were degassed before use. Pentane (Biosolve), diethyl ether and dichloroethane (Merck) were purchased in anhydrous from and stored over MS4 Å after degassing. All other chemicals were purchased from Sigma-Aldrich, Strem, abcr, or TCI. Liquid hydrogenation substrates were degassed and dried by molecular sieves before use. Air and/or moisture sensitive materials were stored in the glovebox. Deuterated solvents were purchased from Eurisotop, dried using molecular sieves, degassed and stored in the glovebox.

NMR spectra were recorded on an Agilent 400-MR DD2 400 MHz spectrometer equipped with a 5 mm ONE NMR probe. All ^{13}C and ^{31}P NMR spectra were recorded with ^{1}H decoupling. All chemical shifts were referenced to residual solvent peaks [$CDCl_3$: 7.26 ppm (^{1}H), 77.2 ppm (^{13}C); $(CD_3)_2SO$: 2.50 ppm (^{1}H), 39.5 ppm (^{13}C); THF-*d8*: 1.72/3.58 ppm (^{1}H), 67.6/25.4 ppm (^{13}C)]. Proton and carbon assignments were made on basis of combined gCOSY and gHSQC spectra. FTIR (both ATR and transmittance modes) was measured on a Bruker Alpha II spectrometer. The solutions of the complexes in THF (0.05 M) were filled in an IR-cell with CaF_2 windows and an optical path length of 0.1 mm which was purged with nitrogen prior to use. The cell was stabilized at 25 °C. The spectra were taken with a resolution of 2 cm^{-1}. Elemental analyses were performed by Mikroanalytisches Laboratorium Kolbe, Oberhausen, Germany.

S2 – Synthetic Procedures and Characterization Data.

Synthesis of Ligand S3:

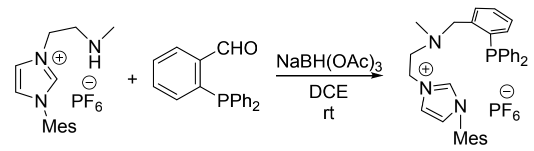

S1 **S2** **S3**

In a Schlenk tube solution of **S1** (1.556 g, 4 mmol) and **S2** (1.218 g, 4.2 mmol) was prepared in dry DCE (20 mL) and stirred overnight. Sodium triacetoxyborohydride (1.336 g, 6.3 mmol) was then slowly added as solid to the resulting solution. After stirring for another night, the solution was quenched by $KHCO_3$ (aq) and extracted with DCM. The combined organic phases were dried over anhydrous Na_2SO_4, concentrated and purified by Et_2O wash to provide the compound **S3** as white foam solid in 49% yield (1.3 g).

^{1}H NMR (400 MHz, THF-d_8, 297 K) δ 8.95 (s, 1H), 7.81 (s, 1H), 7.59 (s, 1H), 7.42 – 7.03 (m, 15H), 6.93 – 6.82 (m, 1H), 4.44 (t, J = 5.8 Hz, 2H), 3.79 (s, 2H), 2.90 (t, J = 5.7 Hz, 2H), 2.34 (s, 3H), 2.19 (s, 3H), 2.05 (s, 6H);

^{31}P{^{1}H}NMR (162 MHz, THF-d_8, 297 K) δ -14.3;

^{13}C {^{1}H}NMR (101 MHz, THF-d_8, 297 K) δ 144.9 ($^1J_{PC}$ 23.2 Hz), 142.6, 139.1, 138.9 ($^2J_{PC}$ 11.1 Hz), 138.2 ($^1J_{PC}$ 15.1 Hz), 136.6, 135.6, 135.4, 135.3, 133.2, 131.2, 131.0 ($^2J_{PC}$ 6.1 Hz), 130.5, 130.4, 130.3, 130.2, 129.0, 125.7, 125.3, 61.8, 58.1, 49.5, 42.5, 22.0, 18.2.

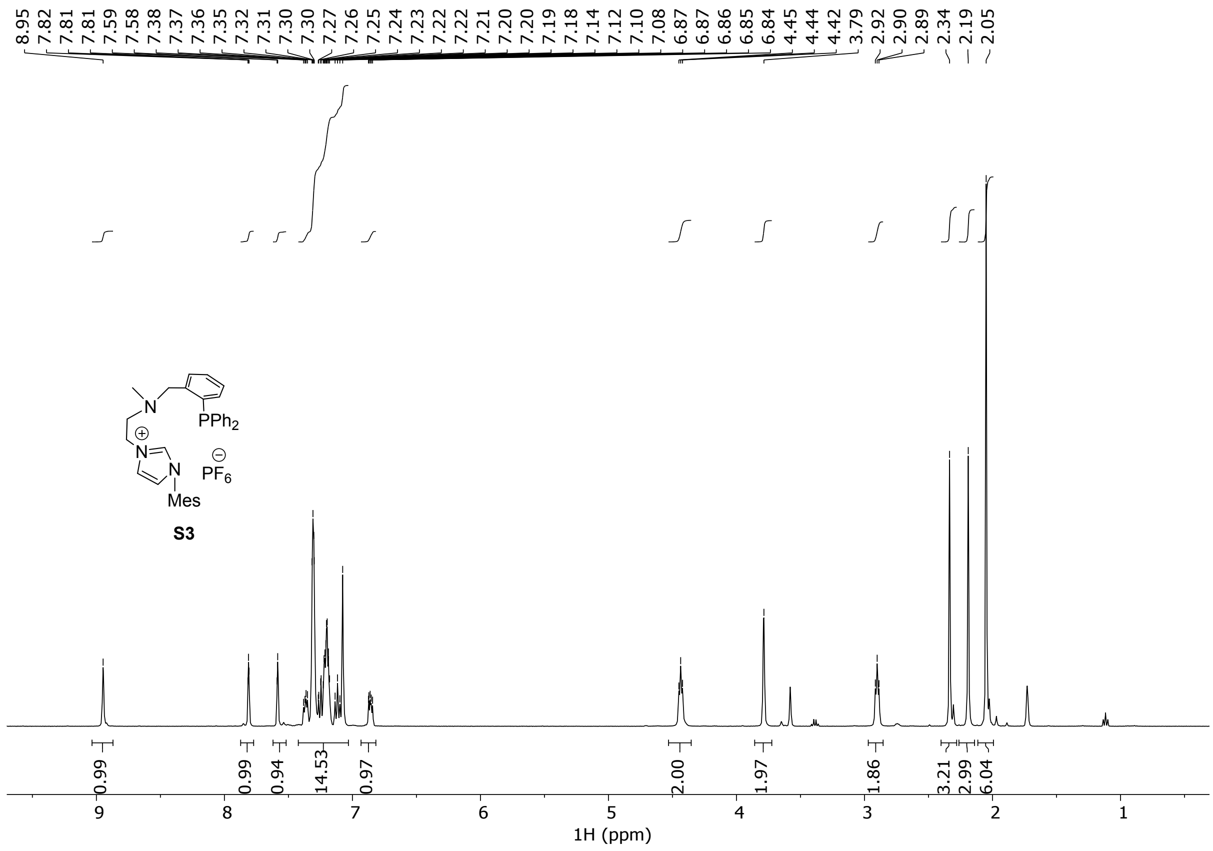

Figure S1. ^{1}H-NMR spectrum of ligand **S3** in THF-d_8 (400 MHz).

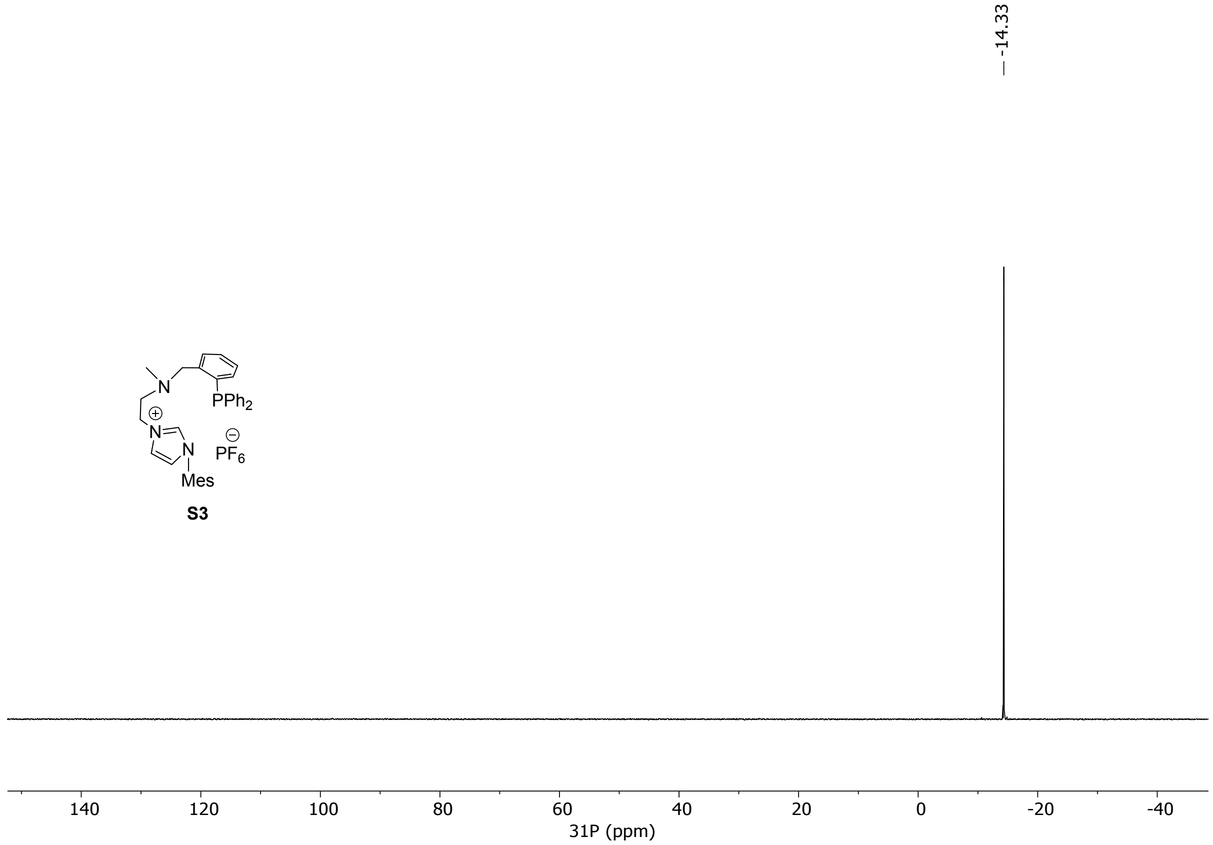

Figure S2. ^{31}P {^{1}H}NMR spectrum of complex **S3** in THF-d_8 (162 MHz).

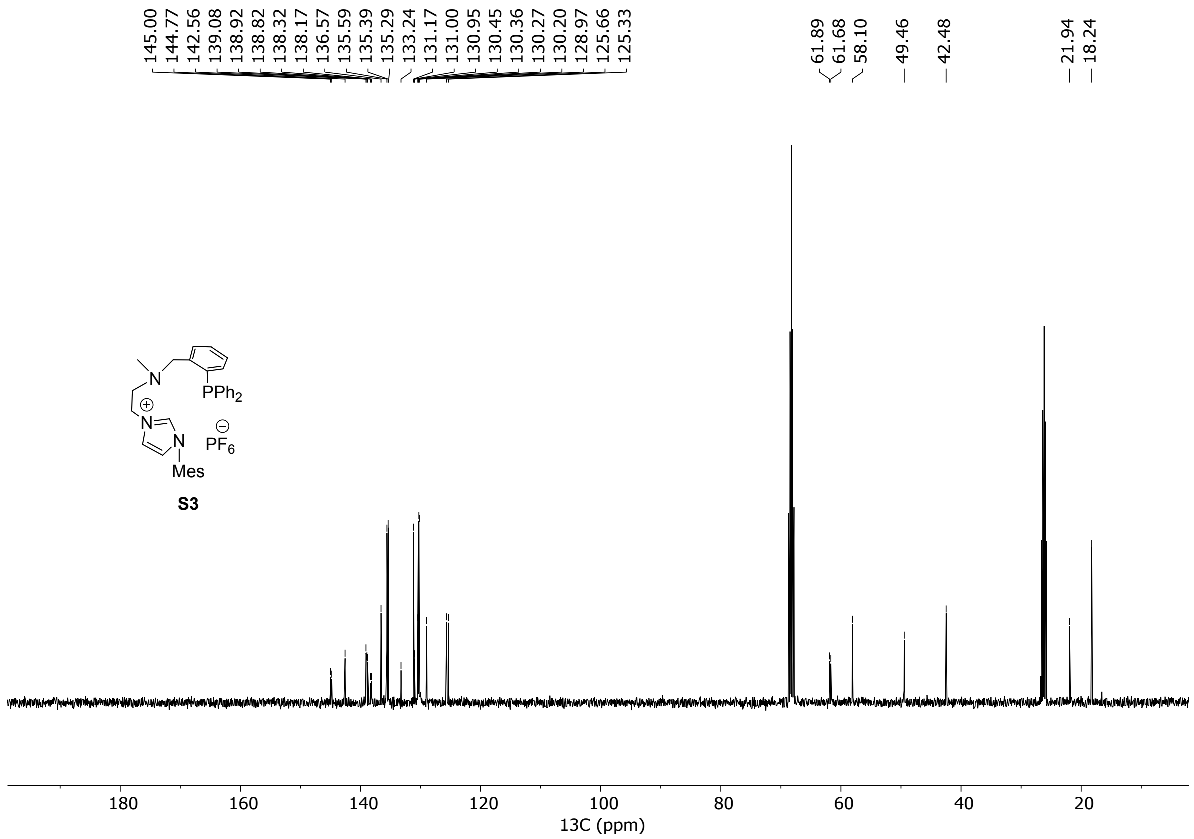

Figure S3. ^{13}C-NMR spectrum of ligand **S3** in THF-d_8 (101 MHz)

Synthesis of complex Mn-7:

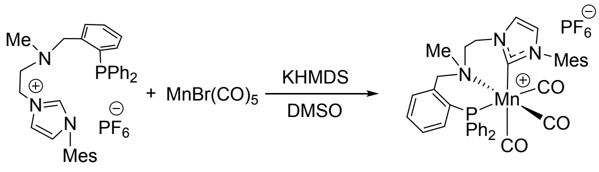

To the solution of ligand **S3** (663 mg, 1 mmol) in DMSO (5 mL) was slowly added KHMDS solution (209.4 mg, 1.05 mmol, in 0.5 mL DMSO) and stirred at rt for 5 min under Ar atmosphere. The $MnBr(CO)_5$ was then added and stirred for another 12 h. The resulting reaction mixture was then quenched by H_2O (15 mL) and extracted by DCM for 3 times. The combined organic phase was dried over $MgSO_4$, filtered, evaporated to dryness and further purified by crystallization (petane vapor diffusion into solution in DCM) as light yellow solid in 57 % yield (460 mg).

Complex **Mn-7**: ^{1}H NMR (400 MHz, CD_2Cl_2, 297 K) δ 7.79 – 7.32 (m, 11H), 7.32 – 7.03 (m, 3H), 6.98 (s, 1H), 6.72 – 6.65 (m, 3H), 4.08 (d, J = 15.2 Hz, 1H), 3.88 (d, J = 12.7 Hz, 1H), 3.48 – 3.35 (m, 2H), 3.05 – 2.87 (m, 6H), 2.22 (s, 3H), 1.95 (s, 3H), 1.73 (s, 3H);

^{31}P{^{1}H}NMR (162 MHz, CD_2Cl_2, 297 K) δ 37.2;

^{13}C {^{1}H}NMR (101 MHz, CD_2Cl_2, 297 K) δ 217.0, 216.6, 214.8, 185.6, 140.3, 137.3 ($^1J_{PC}$ 16.2 Hz), 136.4, 135.7, 133.8 ($^3J_{PC}$ 8.1 Hz), 133.4, 133.3, 132.9 ($^2J_{PC}$ 11.1 Hz), 132.3, 131.8, 131.4, 130.4, 130.0, 129.8 ($^3J_{PC}$ 7.1 Hz), 129.7, 129.6, 129.37, 129.0 ($^2J_{PC}$ 10.1 Hz), 128.4, 128.0 ($^2J_{PC}$ 9.1 Hz), 126.9, 125.6, 63.9, 58.4, 45.5, 20.6, 19.1, 17.5;

IR (solution in THF): ῡ [cm^{-1}] 2022 (s, ῡ CO), 1937 (s, ῡ CO), 1925 (s, ῡ CO);

EA: Found (Calcd.) for $C_{37}H_{36}F_6MnN_3O_3P_2$: C: 55.07 (55.44); H: 4.51 (4.53); N: 5.19 (5.24).

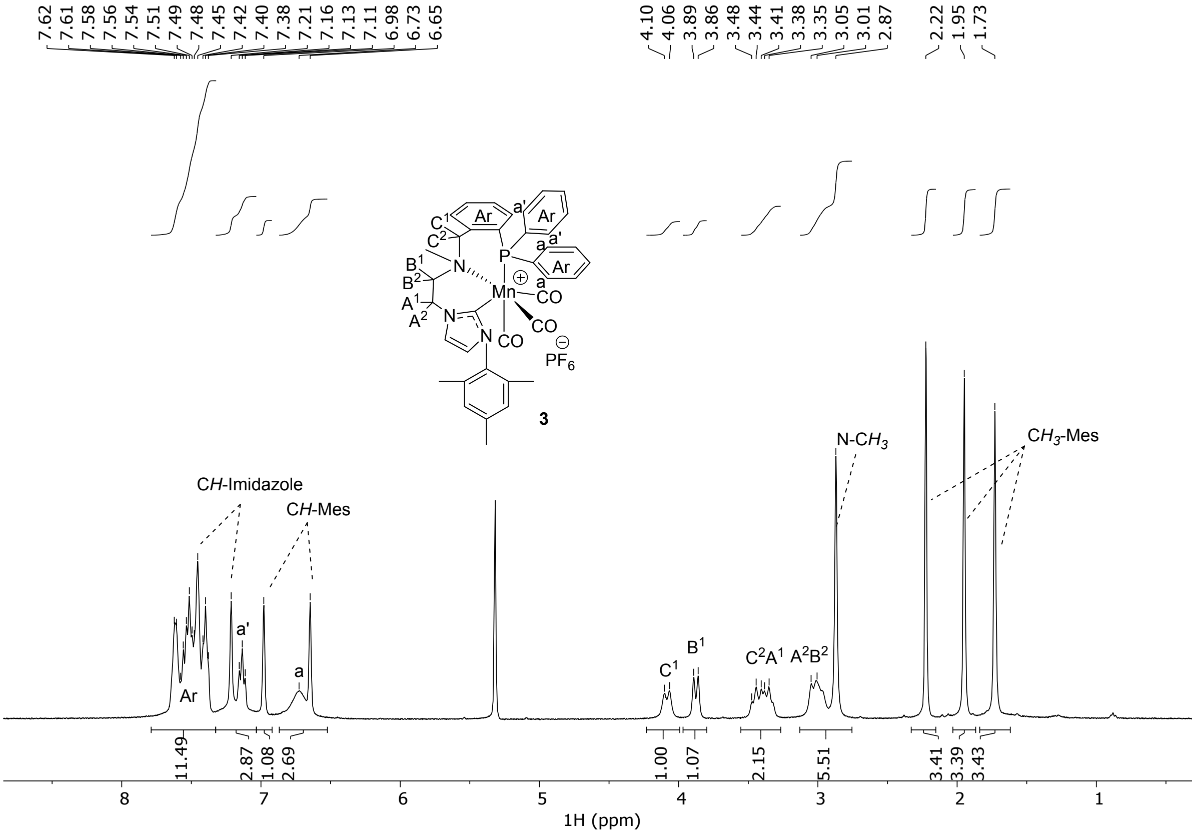

Figure S4. ^{1}H-NMR spectrum of complex **Mn-7** in CD_2Cl_2 (400 MHz).

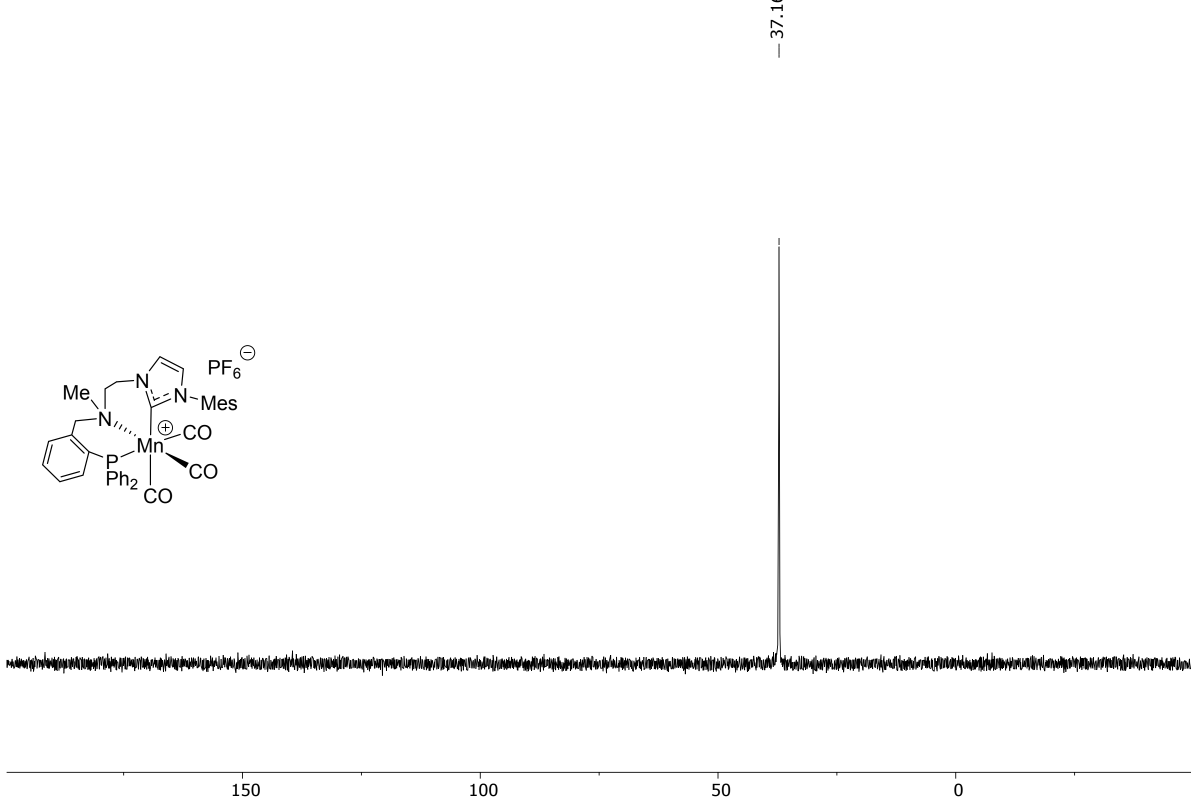

Figure S5. ^{31}P $\{^{1}H\}$NMR spectrum of complex **Mn-7** in CD_2Cl_2 (162 MHz).

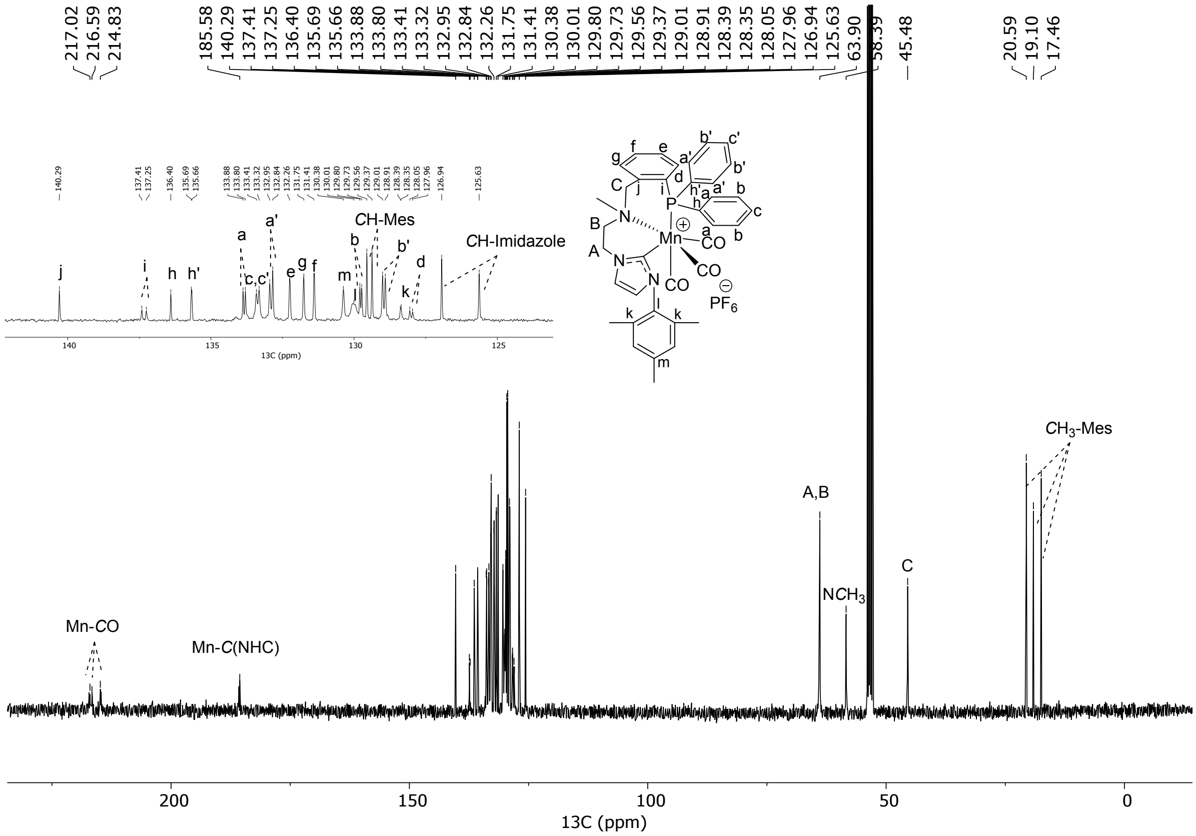

Figure S6. ^{13}C {^{1}H}NMR spectrum of complex **Mn-7** in CD_2Cl_2 (101 MHz).

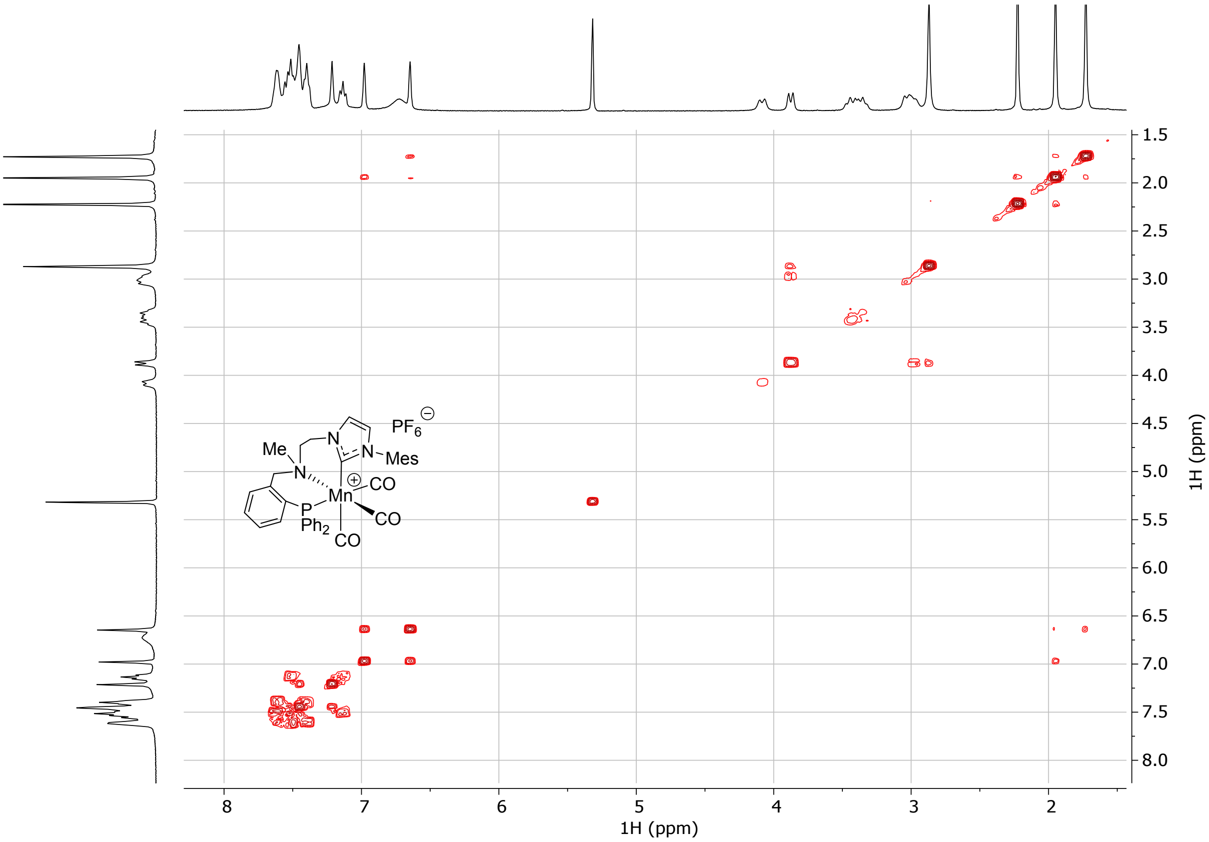

Figure S7. gCOSY spectrum of complex **Mn-7** in CD_2Cl_2.

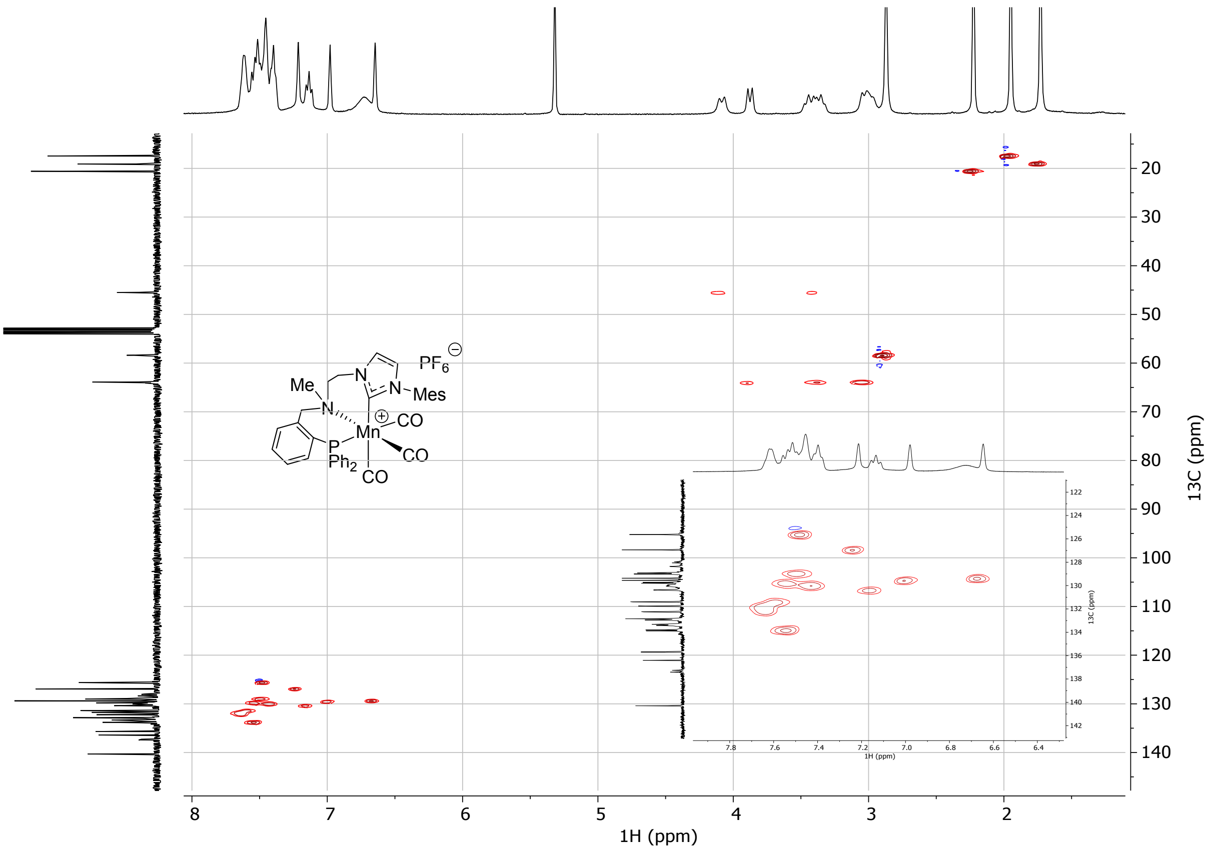

Figure S8. gHMQC spectrum of complex **Mn-7** in CD_2Cl_2.

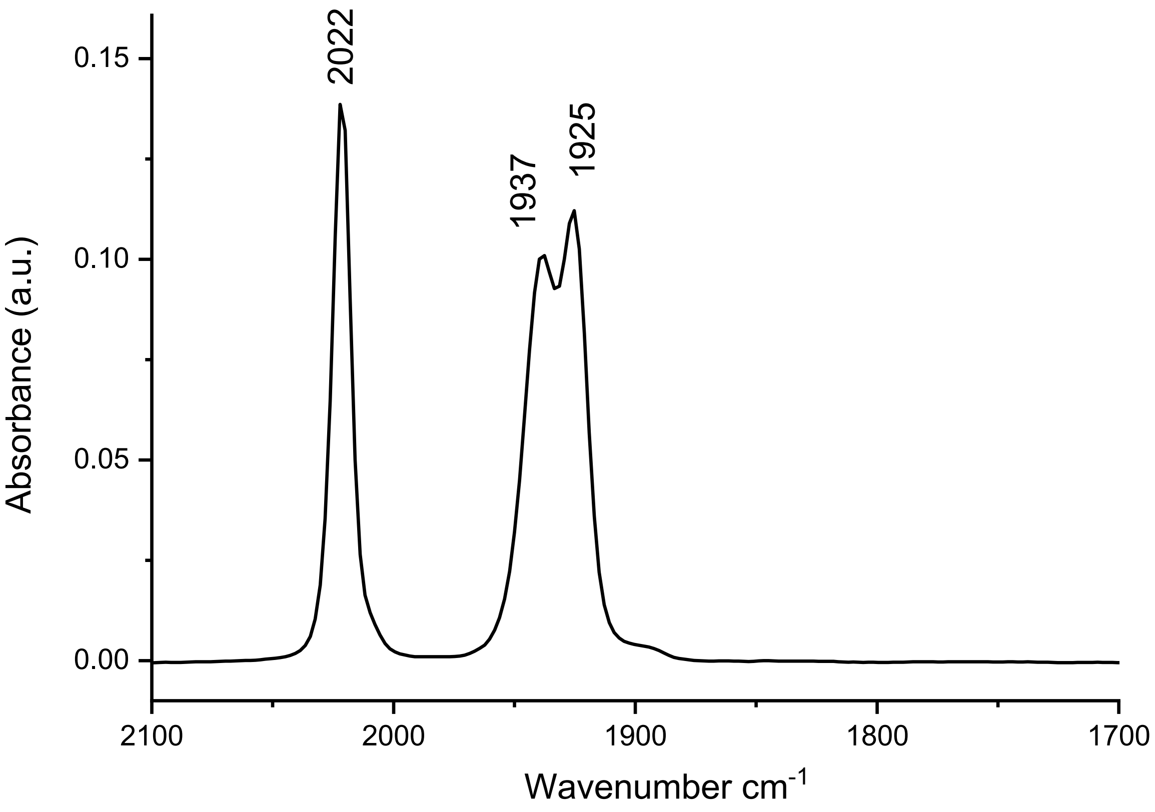

Figure S9. IR spectrum of THF solution of complex **Mn-7.**

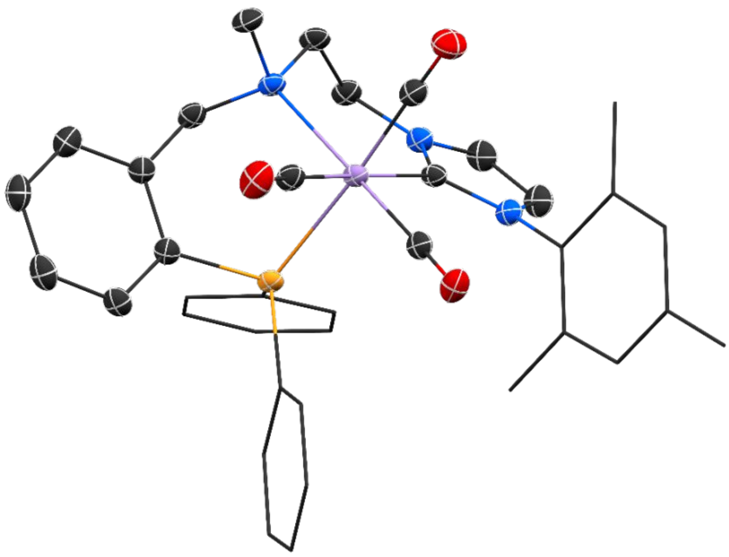

Figure S10. Molecular structure of complex **Mn-7**$^+$ in crystal with thermal ellipsoids drawn at 50% probability (CCDC 2165735).

S3 –Reactivity Study.

In-situ generation of hydride complex via reaction with $KBHEt_3$:

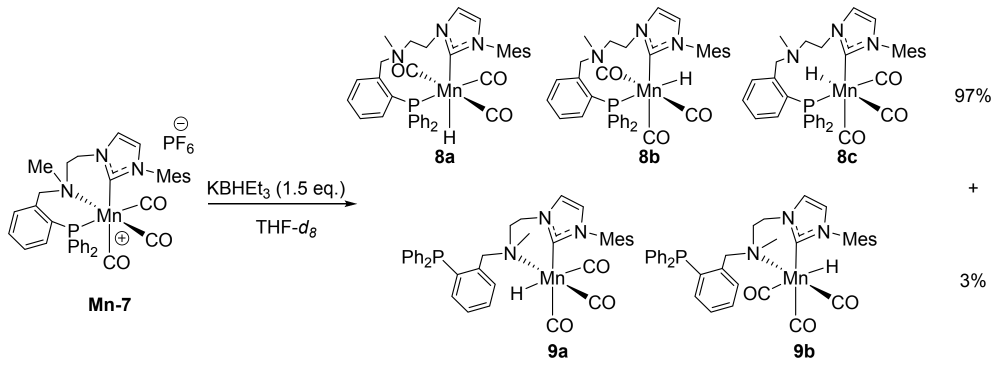

Upon treatment of complex **Mn-7** (8.0 mg, 1 equiv.) with $KBHEt_3$ (15 µl of 1M THF solution, 1.5 equiv.) in THF-d_8 (0.6 mL) an orange solution was formed immediately producing the mixture of hydride complexes **8** and **9** followed by NMR measurements. IR analysis was performed through same procedure with protic THF as solvent. The putative structural assignments of isomers of **8** and **9** are done with the support of DFT calculations shown below in Section S8.

8 (*in situ*): ^{1}H NMR (400 MHz, THF-d_8, 297 K, hydride resonances) δ -5.01 (d, $^2J_{PH}$ = 40.0 Hz), -5.65 (d, $^2J_{PH}$ = 48.0 Hz),.-5.77 (d, $^2J_{PH}$ = 88.0 Hz); ^{31}P{^{1}H}NMR (162 MHz, THF-d$_8$, 297 K): δ = 86.5, 66.1, 59.7; IR (solution in THF, 297 K): ῡ [cm^{-1}] 1989 (s, ῡ CO), 1901 (s, ῡ CO), 1885 (s, ῡ CO).

9 (*in situ*): ^{1}H NMR (400 MHz, THF-d_8, 297 K, hydride resonances) δ -3.50, -3.80; ^{31}P{^{1}H}NMR (162 MHz, THF-d$_8$, 297 K): δ = -12.3, -12.4;

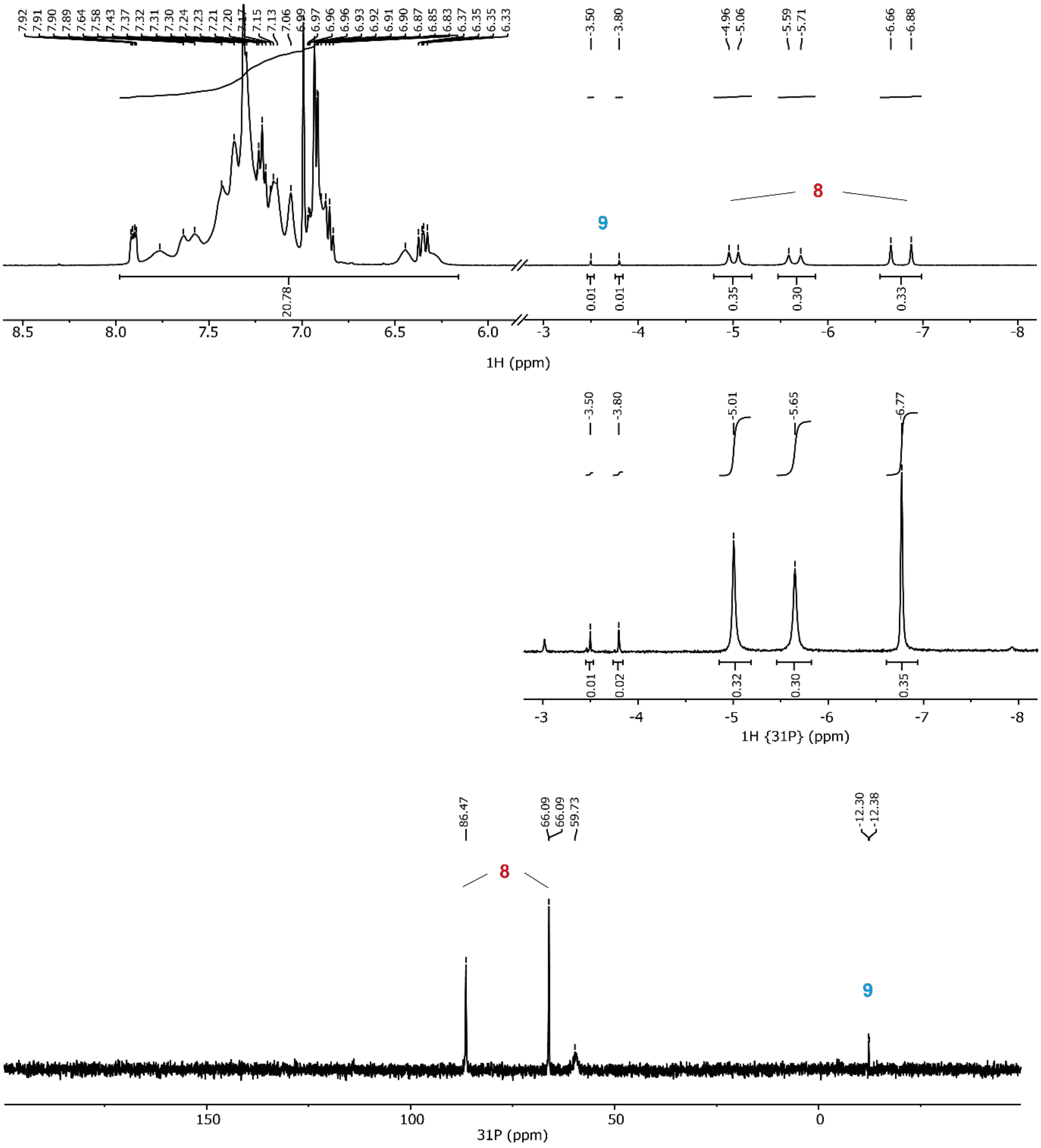

Figure S11. ^{1}H-NMR, ^{1}H{^{31}P}-NMR and ^{31}P-NMR spectrum of $KBHEt_3$-activated **Mn-7** in THF-d_8 immediately after reaction. Bidentate Mn-H **8** with free nitrogen donor were formed as major products. Additionally, hydride from complex **9** with free phosphine donor was also formed in trace amount. The disappearance of phosphine-hydride coupling feature for **8** and the retention of two hydride resonances for **9** in phosphorus decoupled ^{1}H NMR are consistent with the bound and non-bound nature of P-arm in **8** and **9**, respectively. The Integration shown in Figure S22 indicates nearly quantitative conversion to hydride species, and estimates the fraction of **8** at ca. 97% and **9** at ca. 3%.

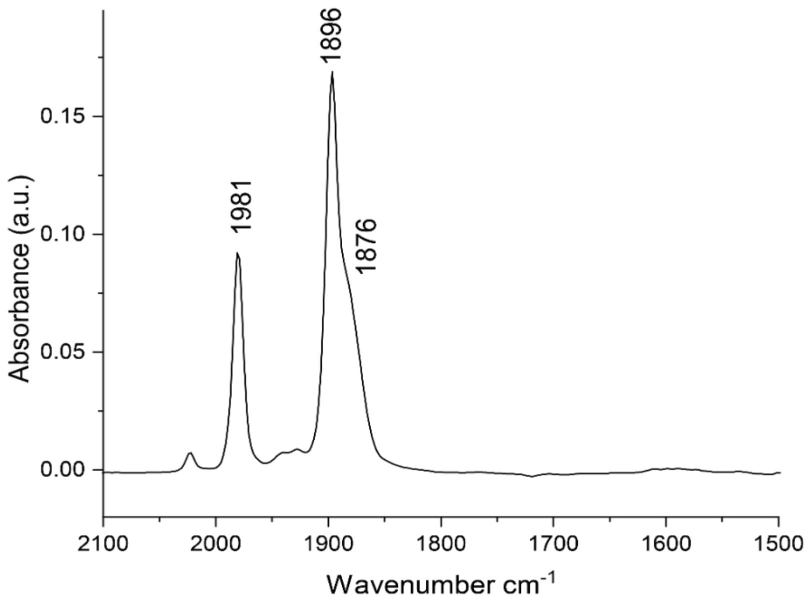

Figure S12. IR spectrum for *in situ* activation of **Mn-7** with $KBHEt_3$ in THF. Note that major species are Mn tricarbonyls.

Reaction of Mn hydride species with alkene:

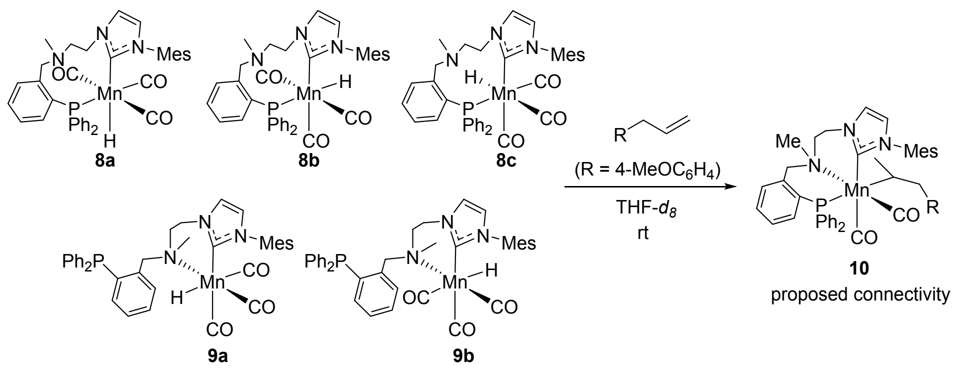

To the mixture of **8** and **9** in THF-d_8 obtained from complex **Mn-7** (Figures S11, 12) was added 4-allylanisole (100 eq.). The Mn hydrides was gradually consumed producing suggested species **10** – an alkyl-bound dicarbonyl complex as suggested by spectroscopy. The same mixture in protic THF was monitored by IR.

10 (*in situ*): $^{31}P\{^{1}H\}$NMR (162 MHz, THF-d_8, 297 K): δ = 43.5, 43.3, 40.8, 37.1; IR (solution in THF, 297 K): $\bar{\upsilon}$ [cm^{-1}] 1903 (s, $\bar{\upsilon}$ CO), 1828 (s, $\bar{\upsilon}$ CO).

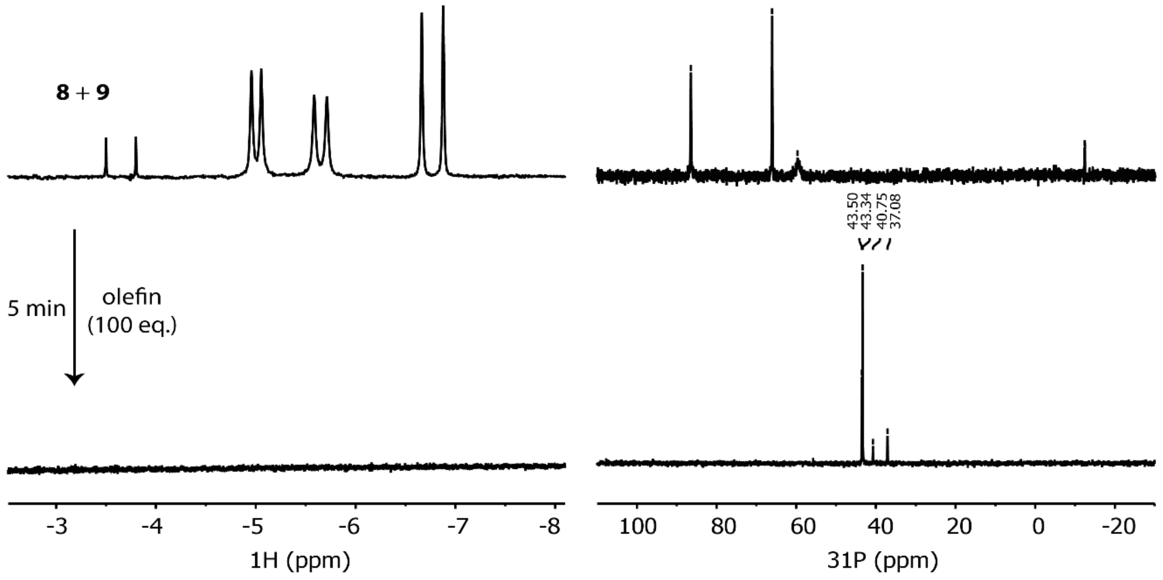

Figure S13. ^{1}H-NMR (hydride region) and ^{31}P-NMRspectrum of in-situ formed mixture of **8** + **9** and its reaction with excess olefin in THF-d_8. The hydrides are fully consumed by olefin, resulting the mixture of stable Mn-alkyl species.

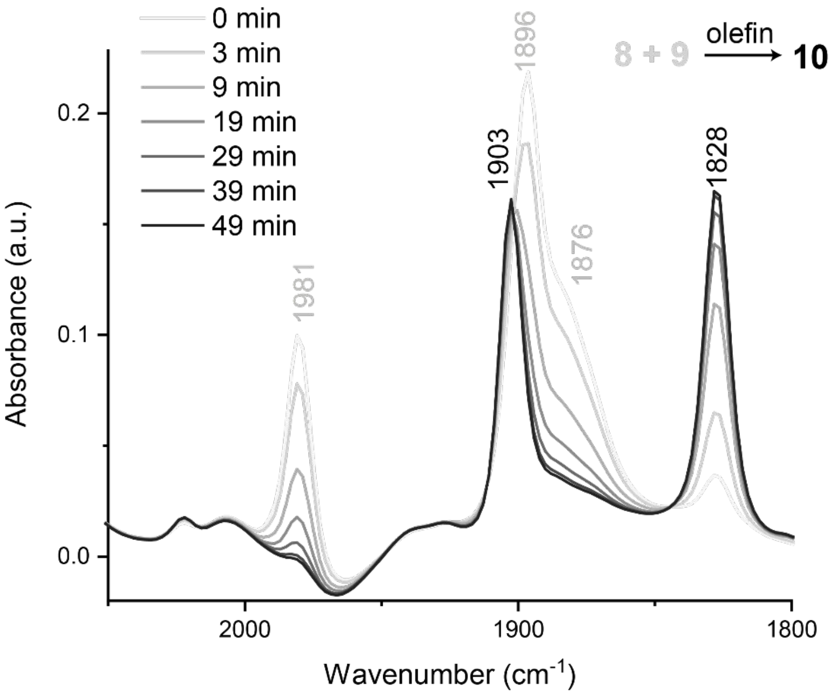

Figure S14. IR spectrum evolution of the reaction between Mn hydrides (**8** and **9**) with excessive olefin for 0 min, 3 min, 9 min, 19 min, 29 min, 39 min, and 49 min reaction times. Note the decrease of three IR bands of complex **8+9** along with the increase of two bands of complex **10** (1903, 1828 cm^{-1}) indicating conversion of **8+9** to **10** via the loss of one CO ligand.

Kinetic tracking of alkene transposition by NMR:

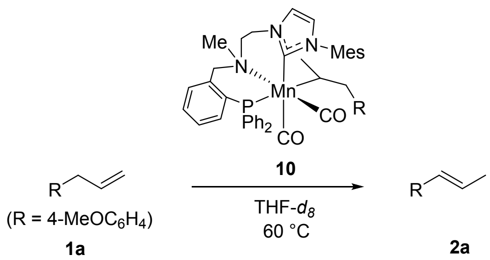

The mixture of Mn **10** prepared as above and 4-allylanisole was heated to 60 °C and monitored by NMR. While allylanisole was converted to 2-alkene, catalyst **10** remained unchanged throughout the reaction. The latter indicate that species **10** should be the resting state for the catalytic cycle.

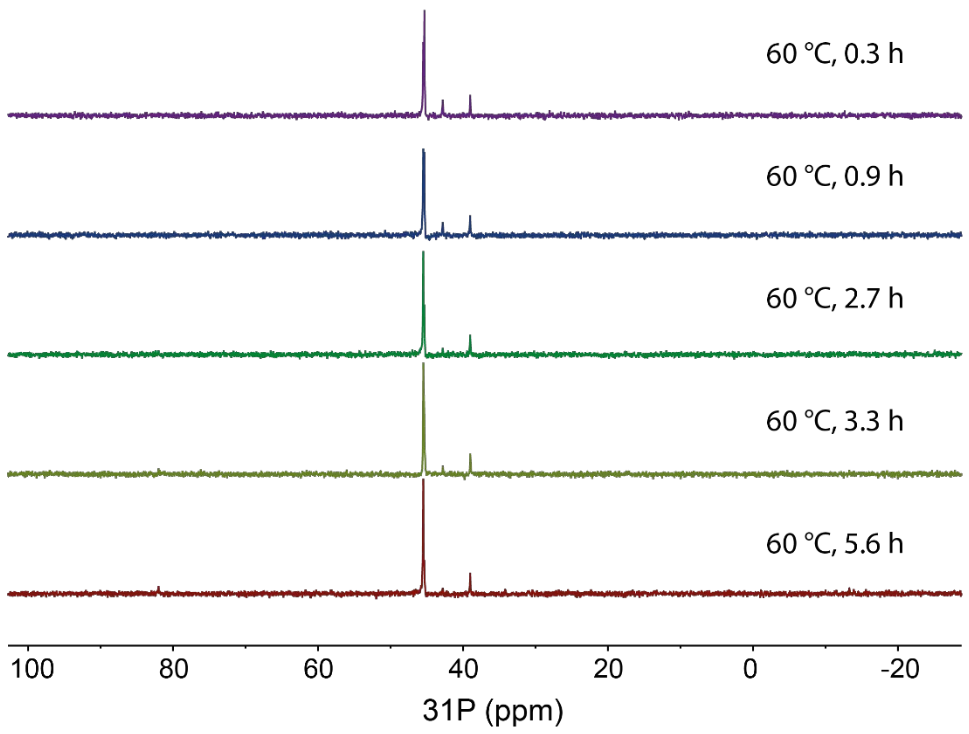

Figure S15. ^{31}P-NMR spectrum evolution of Mn **10** in the course of isomerization of **1a** at 60 °C in THF-d_8.

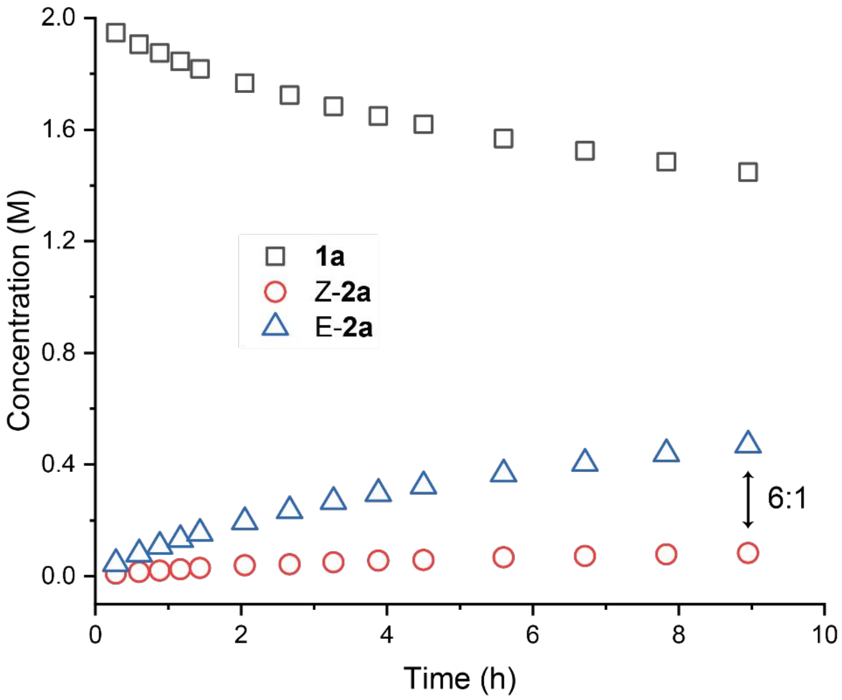

Figure S16. Kinetic trace of Mn **10**-promoted isomerization of **1a** at 60 °C in THF-d_8.

Determination of reaction order of the olefin substrate:

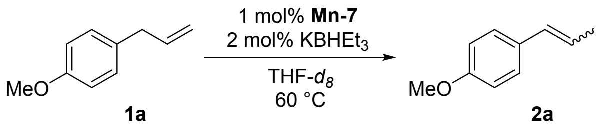

Inside glovebox, solution of **Mn-7** (2.0 mg, 1 mol%) and internal standard (0.25 mmol) was prepared in 0.5 mL THF-d_8 and activated with $KBHEt_3$ (5 μL, 2.0 mol%, 1M THF solution) in a *J*-Young NMR tube for 5 min. Substrate **1** (0.35, 0.30, 0.25, 0.20 mmol) was added, and the reaction mixture was subsequently tracked by NMR at 60 °C.

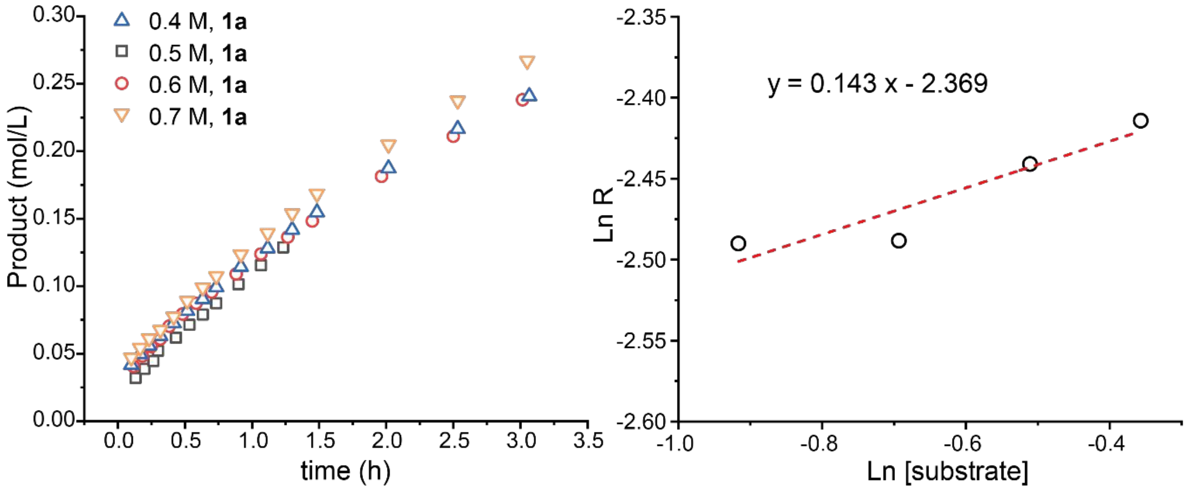

Figure S17. Determination of reaction order. Left: kinetic trace of isomerization reactions of olefin **1a** at different initial concentrations (0.4, 0.5, 0.6, 0.7 M); Right: the plot of ln (initial reaction rate) v.s. ln (initial substrate concentration). As indicated in the plot, the reaction order of the olefin substrate is 0.143, close to 0.

S4-Deuterium labelling experiments

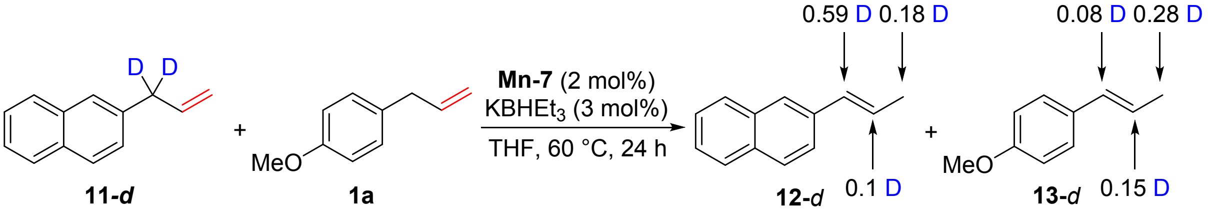

Into a 4 mL brown glass vial was added Mn complex **7** (5.1 mg, 0.0075 mmol), $KBHEt_3$ (3.8 µL, 1 M solution in THF), and THF (0.3 mL). The solution was stirred in glove box at room temperature for 10 mins followed by the addition of substrate **11-*d*** (0.125 mmol, prepared according to reported procedures[1]), substrate **1a** (12 mg, in total 10 mol%), and 0.2 mL THF. The vial was then transferred to oil bath and allowed to stir at 60 °C for 24 h. After the reaction, resulting mixture was cooled to ambient temperature and purified by flash chromatography (pentane) to afford **12-*d*** and **13-*d*** with full conversion.

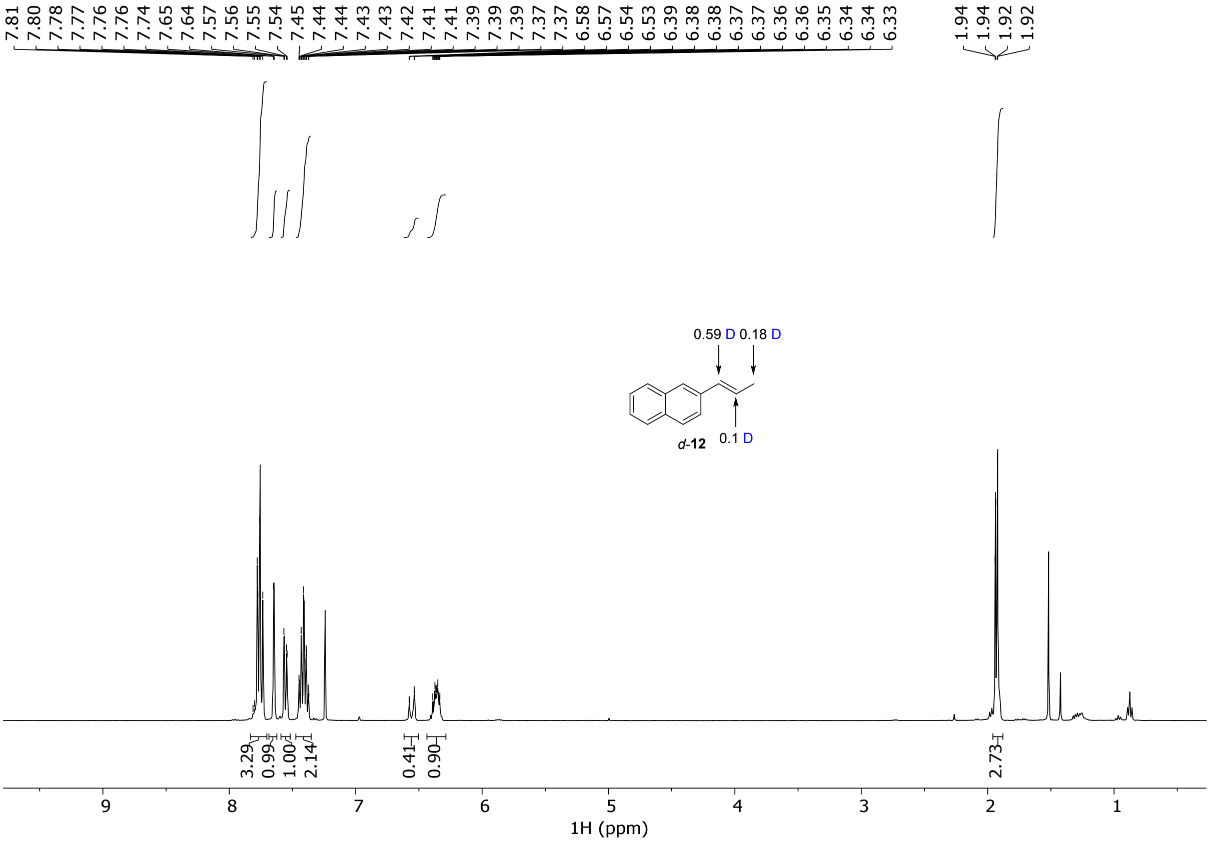

Figure S18. ^{1}H-NMR spectrum of compound **12-*d*** in $CDCl_3$ (400 MHz).

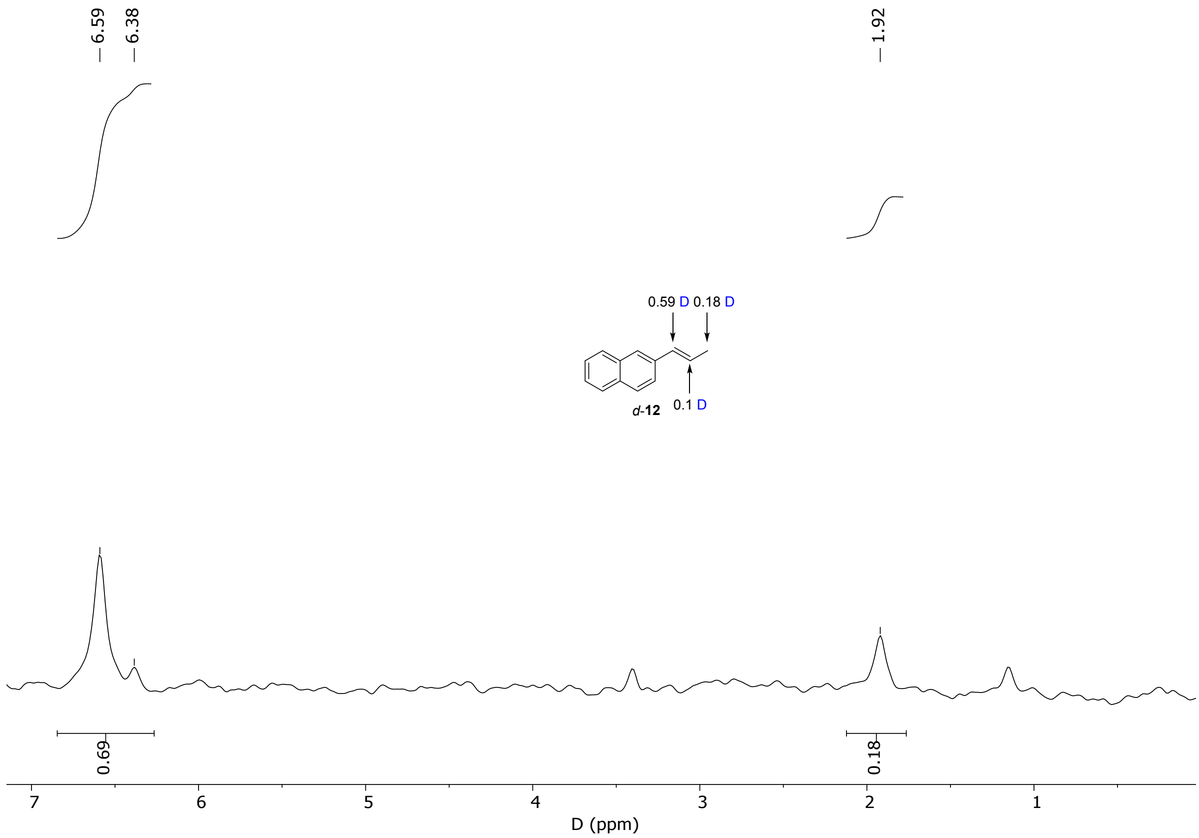

Figure S19. ^{2}H-NMR spectrum of compound **12-*d*** in Et_2O containing 10% $CDCl_3$ (400 MHz).

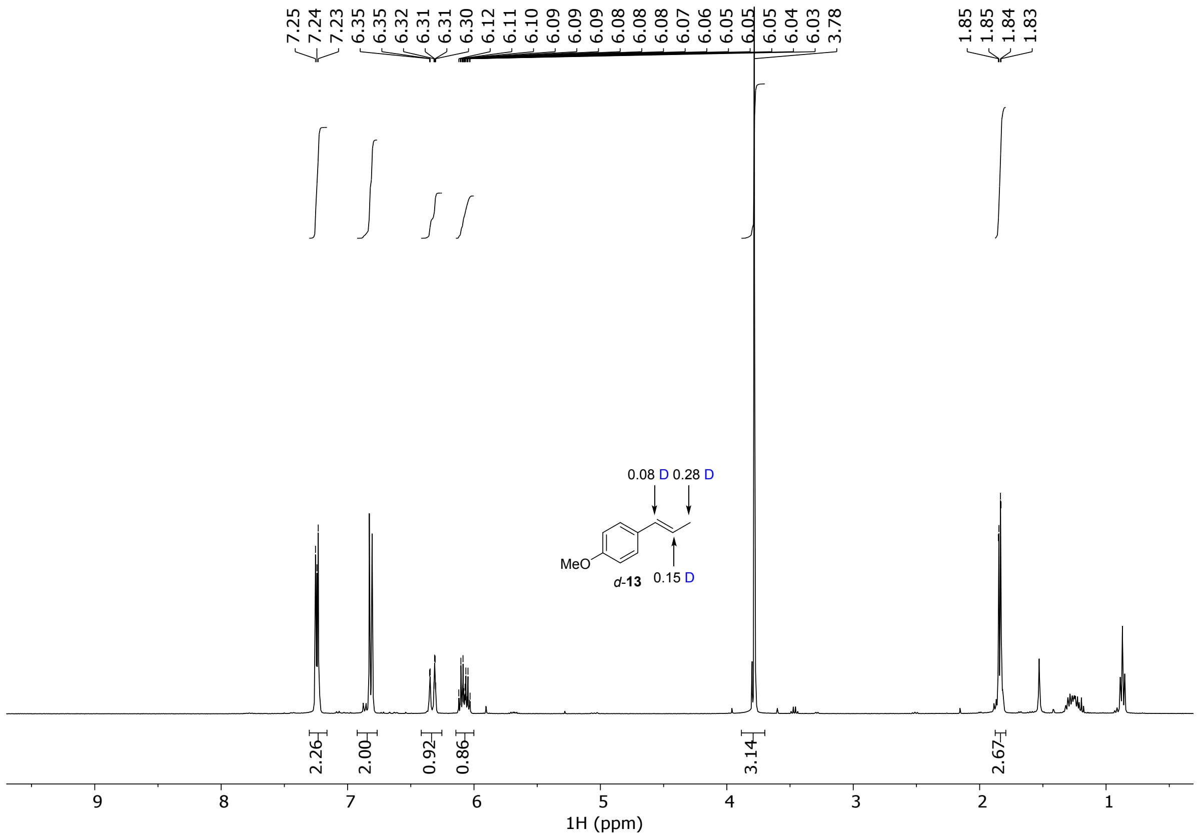

Figure S20. ^{1}H-NMR spectrum of compound **13-*d*** in $CDCl_3$ (400 MHz).

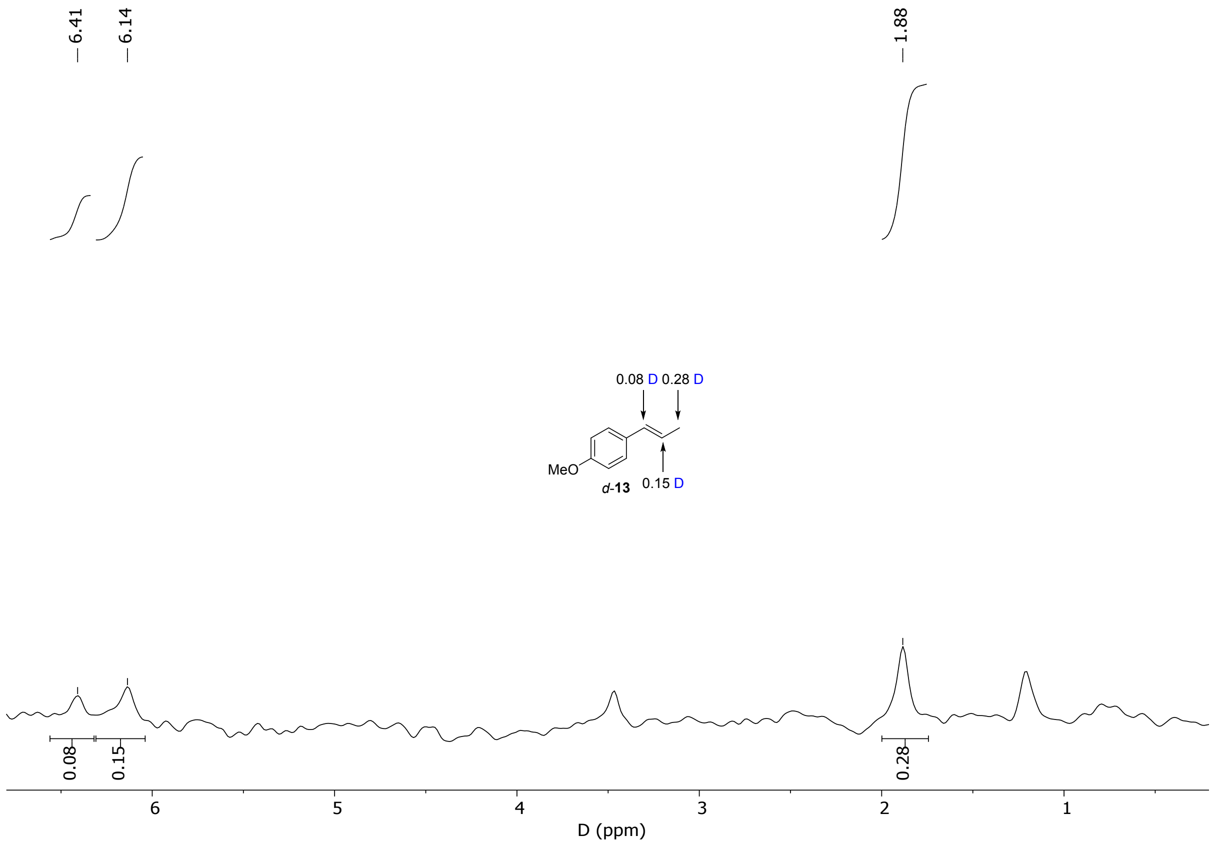

Figure S21. ^{2}H-NMR spectrum of compound **13-*d*** in Et_2O containing 10% $CDCl_3$ (400 MHz).

S5 – Catalysis Details.

General procedure:

All the liquid substrates and internal standard reagents (1-methyl naphthalene) were passed through a plug of neutral alumina, degassed and stored over molecular sieves in the glove box. Mn catalysts, $KBHEt_3$ solution, substrates and solvents were handled in the glovebox. Products were analysed by ^{1}H-NMR. Mass balances were verified to be within 90 % – 110 % for all experiments.

Catalyst Mn-7 was prepared as described above, all other catalysts used in initial screening were prepared according to the published protocols (Mn-3, Mn-4, Mn-5 and Mn-6)[2].

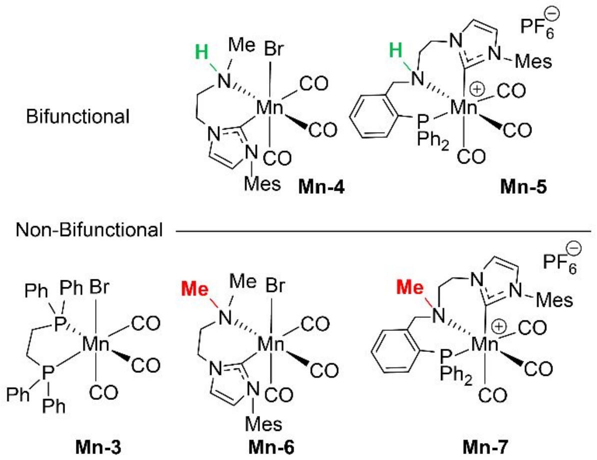

Catalytic transposition of alkenes with Mn-7:

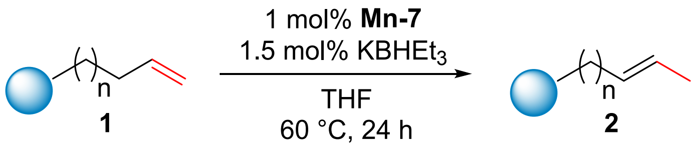

Inside glovebox, solution of **Mn-7** (2.0 mg, 1 mol%) was prepared in 0.3 mL THF and activated with $KBHEt_3$ (4 µL, 1.5 mol%, 1M THF solution) for 10 min. Another solution with substrate **1** (0.25 mmol), THF (0.3 mL), and 1-methyl naphthalene (11.2 µL, 0.0789 mmol) was prepared. 10 µL of substrate mixture was taken and dissolved in $CDCl_3$ as the reference for yield analysis. The catalyst and substrate solutions were combined in a vial as reaction mixture that was subsequently dwelled at 60 °C for 24 h. After the reaction, resulting mixture was cool down to room temperature. NMR samples of products were prepared by the dilution of the reaction mixture in $CDCl_3$ (30 µL into 0.5 mL deuterium solvent). The products were identified and quantified by ^{1}H-NMR.

Replications of catalysis:

To confirm the reproducibility of the catalysis data, several experiments described in Table S1 were triplicated. The results were shown as follows,

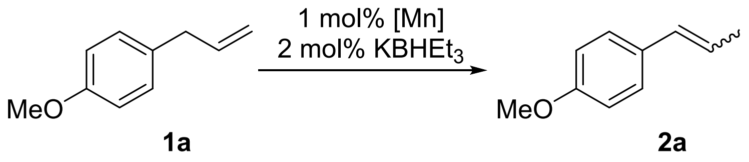

entries	T/°C	solvent	Entries	yield%	Z:E
Group 1	60	THF	1-1	97	11:1
			1-2	99	11:1
			1-3	93	12:1
Group 2	50	THF	2-1	60	7:1
			2-2	70	6:1
			2-3	61	8:1
Group 3	60	Toluene	3-1	75	6:1
			3-2	78	8:1
			3-3	78	8:1
Group 4	60	MeCN	4-1	Trace	-
			4-2	Trace	-
			4-3	trace	-

Reactions were conducted with **1a** (0.25 mmol), **Mn-7** (1 mol%), $KBHEt_3$ (2 mol%) in solvent (0.5 mL) for 24 h

S6 – Optimization of Reaction Conditions.

Table S1. Condition screening for transposition of 4-allylaisole with **Mn-7**.[a]

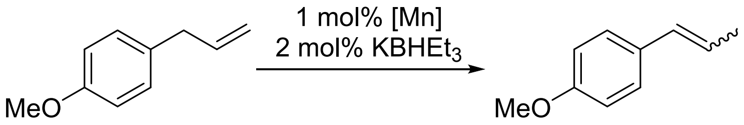

1a **2a**

entries	T/°C	solvent	yield%	Z:E
1[b]	70	THF	36	4:1
2	70	THF	99	>20:1
3	60	THF	97	11:1
4	50	THF	60	7:1
5	40	THF	8	5:1
6	60	DCM	-	
7	60	Toluene	75	6:1
8	60	dioxane	21	6:1
9	60	IPA	59	6:1
10	60	MeCN	-	
11	60	hexane	66	9:1

[a] Reactions were conducted with **1a** (0.25 mmol), **Mn-7** (1 mol%), $KBHEt_3$ (2 mol%) in solvent (0.5 mL) for 24 h. [b] Reaction time was 12 h instead

S7 – Reactivities of Mn complexes toward C=O and C=C.

Catalytic hydrogenation of acetophenone:

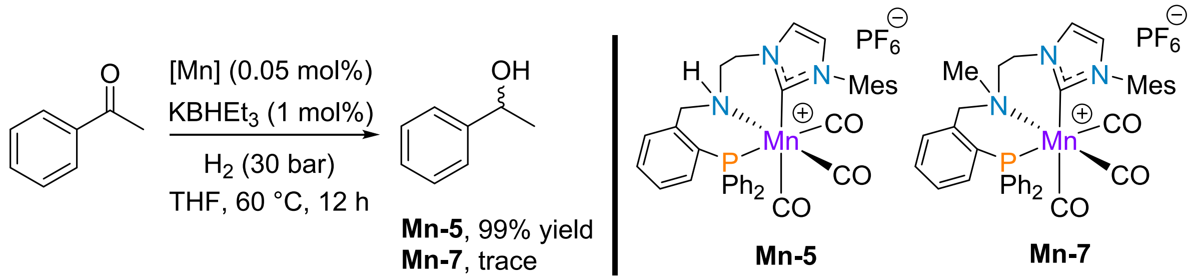

Inside glovebox, the solution of Mn complex (0.05 mol%) was prepared in 1 mL THF and activated with $KBHEt_3$ (10 µL, 0.2 mol%, 1M THF solution) for 10 min. Acetophenone (5 mmol), THF (3 mL), dodecane (56.8 µL, 0.25 mmol) were then added. The resulting mixture was transferred into a stainless steel autoclave in the glovebox. The system was purged with N_2 (3×8 bar) and H_2 (1×30 bar), pressurized with H_2 to 30 bar, and heated to 60 °C. After 12 h, resulting mixture was depressurized, cool down to room temperature, and analysed on an Agilent 6890 gas chromatograph equipped with an FID detector. GC samples were prepared by dilution of the reaction mixture in THF (20 µL into 1 mL THF).

Catalytic hydrogenation of styrene:

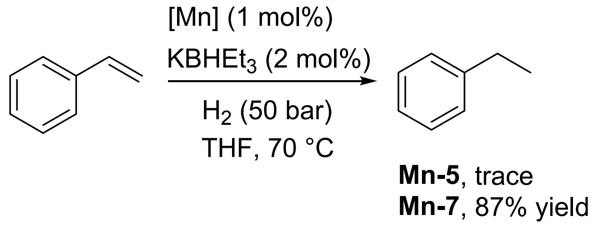

Inside glovebox, the solution of Mn complex (1 mol%) was prepared in 0.2 mL THF and activated with $KBHEt_3$ (5 µL, 2 mol%, 1M THF solution) for 10 min. Styrene (0.25 mmol), THF (0.3 mL), dodecane (56.8 µL, 0.25 mmol) were then added. The resulting mixture was transferred into a stainless steel autoclave in the glovebox. The system was purged with N_2 (3×8 bar) and H_2 (1×30 bar), pressurized with H_2 to 50 bar, and heated to 70 °C. After 12 h, resulting mixture was depressurized, cool down to room temperature, and analysed on an Agilent 6890 gas chromatograph equipped with an FID detector. GC samples were prepared by dilution of the reaction mixture in THF (20 µL into 1 mL THF).

S8 – Computational Studies.

S8.1 Relative stabilities of Mn hydrides with hemilabile N and P donors.

MACE Protocol

The MACE protocol automatically constructs all allowed configurations for the hypothetical 6-coordinated Mn-CN$(CO)_3$H and Mn-CP$(CO)_3$H complexes without using any *a priori* information. The workflow takes the ChemAxon[3] SMILES string of the octahedral complex as input and then generates as output 3D atomic coordinates for all possible configurations of the system, considering both the stereochemistry of the octahedral center and the ligands. All following steps, including the filtration of the identical configurations, enantiomers, and impossible structures, generation of conformers using distance geometry approach, and the following MM relaxation is carried out with in-home RDKit-based software[4].

For each of the considered Mn-CN$(CO)_3$H and Mn-CP$(CO)_3$H complexes MACE algorithm produced 4 distinct coordination geometries at the Mn center (2 *facial* and 2 *meridional*). For each coordination environment 50 conformers were generated with the following pruning of similar structures using a 0.5 RMSD threshold, and 5 with the lowest MM energy were selected for the further QM computations giving it total 20 distinct configurations. The obtained structures were further fully relaxed following by frequency calculations using Gaussian 16 revision C01 program package. The starting configurations of the isomers with lowest energy were converged upon the geometry optimization to identical isomers as confirmed by negligible RMSD and identical spectroscopic characteristics for the respective optimized structures. To confirm the exhaustive nature of the configurational search by MACE, additional configurations were manually constructed based on the optimized geometries, which all converged to the structures identified by the automated algorithm.

Calculations

The following optimization and frequency calculations were carried out at the PBE0-D3/6-311+G(d,p)/PCM(THF) level of theory using the Gaussian16 software[5,6]. The calculated frequencies were scaled with the factor f = 0.9576, which was chosen for best agreement of experimental and calculated data. It is applied to minimize the error of the harmonic approach for the vibration frequency calculation. The Half width of the plotted calculated spectra is 5 cm^{-1}. ^{1}H NMR shielding was computed for the optimized geometries at PBE0/6-311++G(2d,2p) level of theory. Relative stabilities of complexes were calculated as energies normalized to the most stable configuration (Table S2). XYZ files containing optimized geometries for all considered systems are given as a supplement and posted in the raw dataset.

Conformational entropies were calculated using two approaches. Firstly, the MACE protocol was used to generate 200 conformations for the most stable C,N- and C,P-bi-bound Mn hydride complexes. The set of unique conformers was obtained based on the values of dihedral angles of all rotatable bonds.

After DFT computations, obtained free energies were used to find conformational entropy S_{conf} using the formula:

$$S_{conf} = -R \cdot \Sigma\, p_i \cdot \ln p_i\,,$$

where p_i is the probability of a complex being in i^{th} conformation, which was found from the Boltzmann distribution. Secondly, the CREST software[7] was used. Several tests showed that semi-empirical GFN2-xTB and molecular mechanical GFN-FF levels of theory poorly reproduce the geometry of the studied complexes. To partially solve this problem, several modifications were applied to the GFN-FF force field for both C,N- and C,P- complexes. The resulting conformational entropy was found as an interval of obtained values (Table S2).

Results and discussion:

MnCNmeP_biN_3 and **MnCNmeP_biP_1** are found as the most stable hydride isomers. They featured hemilabile P and N respectively, of which the structures are shown in Figure S22. The geometries for the other computed structures can be found in attached XYZ files.

As depicted in Figure S22, Mn-CP hydride (with free N) froms the most stable configuration that is preferred over Mn-CN hydride (with free P) by 9 kJ·mol^{-1}. This stability difference is close to that observed in NMR experiments (see Figure S11) suggesting that complexes 8 are dominating the reaction mixture. Therefore, we conclude that hemilability of the central nitrogen atom is apparently persistent for activated **Mn-7**.

Table S2. Relative electronic and free energies of representative bidentate stereomers of Mn hydride complexes.

Free donor group	System[a]	Geometry	Relative energy[b] kJ·mol^{-1}		Conformational entropy $-T\Delta S_{conf}$	
			ΔE	ΔG	QM	CREST
Free **P**	MnCNmeP_biN_0	N-Mn-H anti	51.0	38.8		
	MnCNmeP_biN_1	NMe/MnH syn	45.2	33.6		
	MnCNmeP_biN_2	C-Mn-H anti	45.8	29.7		
	MnCNmeP_biN_3 (conf5)	NMe/MnH anti	20.3	8.8	-1.2	[-9.4 ... -13.6]
Free **N**	MnCNmeP_biP_0	C-Mn-H anti	20.6	18.1		
	MnCNmeP_biP_1	CO-Mn-H anti	0.0	0.0	-1.7	[-12.7 ... -19.2]
	MnCNmeP_biP_2	P-Mn-H anti	11.5	7.8		

	MnCNmeP_biP_3	P-Mn-C anti	37.7	34.4		

a) Names of metal complexes correspond to the names in the comment line of XYZ-files. b) QM energies normalized to the the most stable configuration.

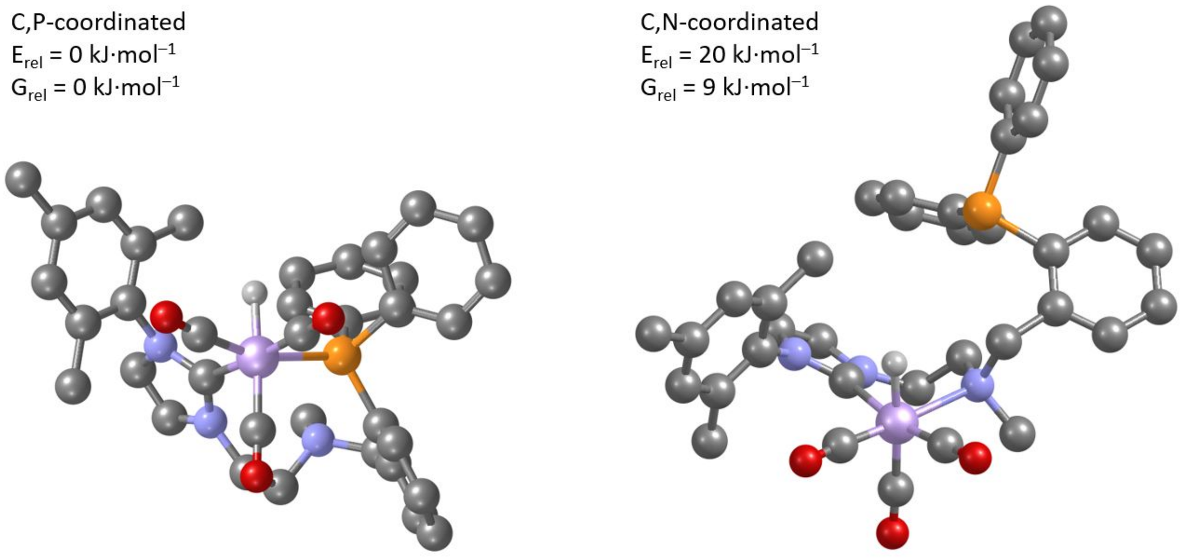

Figure S22. The most stable configurations of C,N- and C,P- coordinated bidentate Mn hydride complexes.

S8.2 Comparison of experimental and calculated spectra for precatalyst avtivation.

While the identification of exact stereochemistry for the isomers of **8** might present a challenge our calculations confirm the ligand connectivity in Mn-**7**, **8**, **9** and the impact of structural differences on NMR and IR spectra.

IR: Conversion of tricarbonyl **7** to tricarbonyl **8** is associated with notable shift of three CO bands that was observed experimentally and confirmed by theory. See table S3 below for comparison of CO vibrational frequencies for CNP and CP-bound complexes.

Table S3. Comparison of theoretical and experimental IR bands for CO stretching of complex **8**.

Vibration mode	IR frequency, cm–1		
	DFT	norm	exp
**Mn-7**, nosymm_1	2043	1936	1925
**Mn-7**, nosymm_2	2049	1941	1937
**Mn-7**, symm	2127	2015	2022
**8**, nosymm_1	1992	1887	1876
**8**, nosymm_2	1994	1889	1896
**8**, symm	2080	1970	1981
Norm. coef.	0.9473		
RMSE	8.83		

NMR: The bidentate Mn species **8** and **9** also show significant differences in computed NMR spectra that are in line with experimental observations. As significant downfield shift was observed for species **9** with free P donor arm. While experimental evidence for P or N dissociation is unambiguous, the agreement with theory further confirms the adequacy of selected assignments.

Table S4. Comparison of theoretical and experimental ^{1}HNMR (hydride) chemical shifts of complexes **8** and **9**.

Complex	Experiment hydride (ppm)	Calculated hydride (ppm)
**8**	-5.01, -5.65, -5.77	-5.7
**9**	-3.50, -3.80	-3.7

S9 – Crystal Structure Analysis Details.

X-ray crystal structure determination of complex Mn-7 :

Crystals suitable for X-ray diffraction were obtained by slow pentane vapour diffusion into mixture THF solution of **Mn-7**. Crystallographic data: $C_{37}H_{36}F_6MnN_3O_3P_2$, C_4H_8O, *Fw* =873.67 g mol^{-1}, light yellow block, 0.30x0.13x0.07 mm^3 (crystal size), monoclinic, *C2/c*, a = 36.7147(3) Å, b = 14.10553(9) Å, c = 17.3980(1) Å, α = 90 ° , β = 115.9524(2) ° , γ = 90 ° , V = 8101.49(10) Å^3, Z = 8, Dx = 1.433 g cm^{-3}, μ = 4.029 mm^{-1}. 7649 reflections were measured by a Bruker D8-Venture Photon area detector (CuKα radiation, λ = 1.54178 Å^{-1}) up to a resolution of (sin(Θ)/ λ) = 0.63 Å^{-1} at a temperature of 100 K.

Data analysis:

Reflections were corrected for adsorption and scaled on the basis of multiple measured reflections using the SADABS program (0.358 – 0.631 correction range).[8] 6435 Reflections were unique (Rint = 0.060). Using ShelXle[9], the structures were solved with SHELXS-18 by using direct methods and refined with SHELXL-2018[10] on *F*2 for all reflections. Non-hydrogen atoms were refined by using anisotropic displacement parameters. Positions of hydrogen atoms were calculated for idealized positions. 518 Parameters were refined without restraints. $R1$ = 0.037 for 7649 reflections with $I > 2\sigma(I)$ and $wR2$ = 0.098 for 8265 reflections. S = 1.077. Residual electron density was between 0.55 and -0.46 eÅ^{-3}. Geometry calculations and checks for higher symmetry were performed with the PLATON program.[11]

CCDC-2165735: complex **Mn-7** contains the supplementary crystallographic data for this paper. These data can be obtained free of charge from The Cambridge Crystallographic Data Centre *via* http://www.ccdc.cam.ac.uk/data_request/cif.

S10 – References.

1. Yu, X.; Zhao, H.; Li, P.; Koh, M. J., Iron-catalyzed tunable and site-selective olefin transposition. J. Am. Chem. Soc. **2020,** 142, 18223-18230

2. For **Mn-4** and **Mn-6** see van Putten, R.; Benschop, J.; de Munck, V. J.; Weber, M.; Müller, C.; Filonenko, G. A.; Pidko, E. A., Efficient and Practical Transfer Hydrogenation of Ketones Catalyzed by a Simple Bidentate Mn− NHC Complex. ChemCatChem **2019**, 11, 5232-5235; **Mn-5** see Yang, W.; Chernyshov, I. Y.; van Schendel, R. K.; Weber, M.; Müller, C.; Filonenko, G. A.; Pidko, E. A., Robust and efficient hydrogenation of carbonyl compounds catalysed by mixed donor Mn (I) pincer complexes. Nat. Commun. **2021,** 12, 1-8; **Mn-3** preparation identical to Weber, S.; Stöger, B.; Kirchner, K., Hydrogenation of nitriles and ketones catalyzed by an air-stable bisphosphine Mn (I) complex. Org. Lett. **2018,** 20, 7212-7215.

3. http://www.chemaxon.com/

4. https://www.rdkit.org/

5. Frisch, M. J.; Trucks, G. W.; Schlegel, H. B.; Scuseria, G. E.; Robb, M. A.; Cheeseman, J. R.; Scalmani, G.; Barone, V.; Petersson, G. A.; Nakatsuji, H.; Li, X.; Caricato, M.; Marenich, A. V; Bloino, J.; Janesko, B. G.; Gomperts, R.; Mennucci, B.; Hratchian, H. P.; Ortiz, J. V; Izmaylov, A. F.; Sonnenberg, J. L.; Williams-Young, D.; Ding, F.; Lipparini, F.; Egidi, F.; Goings, J.; Peng, B.; Petrone, A.; Henderson, T.; Ranasinghe, D.; Zakrzewski, V. G.; Gao, J.; Rega, N.; Zheng, G.; Liang, W.; Hada, M.; Ehara, M.; Toyota, K.; Fukuda, R.; Hasegawa, J.; Ishida, M.; Nakajima, T.; Honda, Y.; Kitao, O.; Nakai, H.; Vreven, T.; Throssell, K.; Montgomery Jr., J. A.; Peralta, J. E.; Ogliaro, F.; Bearpark, M. J.; Heyd, J. J.; Brothers, E. N.; Kudin, K. N.; Staroverov, V. N.; Keith, T. A.; Kobayashi, R.; Normand, J.; Raghavachari, K.; Rendell, A. P.; Burant, J. C.; Iyengar, S. S.; Tomasi, J.; Cossi, M.; Millam, J. M.; Klene, M.; Adamo, C.; Cammi, R.; Ochterski, J. W.; Martin, R. L.; Morokuma, K.; Farkas, O.; Foresman, J. B.; Fox, D. J. Gaussian16 Revision C.01. 2016.

6. Marenich, A. V.; Cramer, C. J.; Truhlar, D. G., Universal solvation model based on solute electron density and on a continuum model of the solvent defined by the bulk dielectric constant and atomic surface tensions. The Journal of Physical Chemistry B 2009, 113, 6378-6396.

7. Pracht, P., Bohle, F., Grimme S. Automated exploration of the low-energy chemical space with fast quantum chemical methods *Phys. Chem. Chem. Phys.*, 2020, 22, 7169-7192

8. Bruker **(2013)**. *APEX2*, *SAINT, XPREP* and *SADABS*. Bruker AXS Inc., Madison, Wisconsin USA.

9. B. Hübschle, G. M. Sheldrick, D Dittrich, ShelXle: a Qt graphical user interface for SHELXL. *J. Appl. Cryst.* **2011**, *44*, 1281 – 1284.

10. G. M. Sheldrick, SHELXT - Integrated space-group and crystal-structure determination. *Acta Cryst.* **2015**, *C71*, 3-8.

11. *PLATON*. A. L. Spek, Structure validation in chemical crystallography. *Acta Cryst.* **2009**, *D65*, 148–155.
